# Guanidinium Substitution Improves Self-Healing and Photodamage Resilience of MAPbI_3_

**DOI:** 10.1021/acs.jpcc.4c06090

**Published:** 2024-11-20

**Authors:** Pallavi Singh, Davide Raffaele Ceratti, Yahel Soffer, Sudipta Bera, Yishay Feldman, Michael Elbaum, Dan Oron, David Cahen, Gary Hodes

**Affiliations:** 1 Dept. of Molecular Chem. & Materials Science, Weizmann Institute of Science, Rehovot 7610001, Israel; 2 CNRS, Chimie ParisTech, Institut de Recherche de Chimie Paris, Physical Chemistry of Surfaces Group, PSL University, 11 rue Pierre et Marie Curie, Paris 75005, France; 3 Dept. of Physics of Complex Systems, Weizmann Institute of Science, Rehovot 7610001, Israel; 4 Dept. of Chemical Research Support, Weizmann Institute of Science, Rehovot 7610001, Israel; 5 Dept. of Chemical & Biological Physics, Weizmann Institute of Science, Rehovot 7610001, Israel

## Abstract

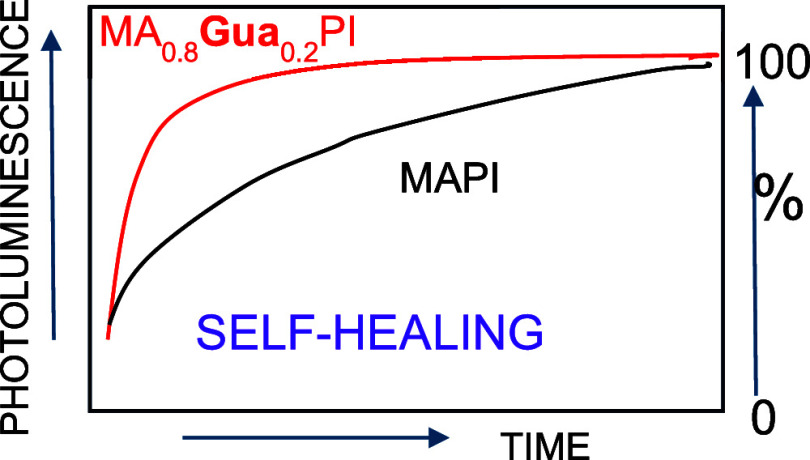

Self-healing materials can become game changers for developing sustainable (opto)electronics. APbX_3_ halide (=X^–^) perovskites, HaPs, have shown a remarkable ability to self-heal damage. While we demonstrated self-healing in pure HaP compounds, in single crystals, and in polycrystalline thin films (as used in most devices), HaP compositions with multiple A^+^ (and X^–^) constituents are preferred for solar cells. We now show self-healing in mixed A^+^ HaPs. Specifically, if at least 15 atom % of the methylammonium (MA^+^) A cation is substituted for by guanidinium (Gua^+^) or acetamidinium (AA^+^), then the self-healing rate after damage is enhanced. In contrast, replacing MA^+^ with dimethylammonium (DMA^+^), comparable in size to Gua^+^ or AA^+^, does not alter this rate. Based on the times for self-healing, we infer that the rate-determining step involves short-range diffusion of A^+^ and/or Pb^2+^ cations and that the self-healing rate correlates with the strain in the material, the A^+^ cation dipole moment, and H-bonding between A^+^ and I^–^. These insights may offer clues for developing a detailed self-healing mechanism and understanding the kinetics to guide the design of self-healing materials. Fast recovery kinetics are important from the device perspective, as they allow complete recovery in devices during operation or when switched off (LEDs)/in the dark (photovoltaics).

## 1 Introduction

Self-healing, in materials, i.e., the ability to autonomously recover from damage, has been mainly investigated in organic polymers.^1^ Till recently, only few examples of self-healing in crystalline inorganic materials were known, very few of semiconductors. These include CuInSe_2_,^2^ the parent material of Cu(In,Ga)Se_2_, used in photovoltaic “CIGS” modules, and Li-doped Si, Si:Li,^3^ used for radiation detection.

Self-healing has recently been demonstrated in lead halide perovskite (HaP) materials^4−11^ and devices^12−15^ made from them. Because the long-term stability of HaP-based devices is a major issue for their commercialization, it is of both fundamental interest and practical importance to understand what enables self-healing in HaP materials themselves.

Earlier, we studied the recovery kinetics in the bulk of single crystals of bromide perovskites,^4,6,11^ of methylammonium lead iodide,^5^ MAPbI_3_ (MAPI), and of 2D and 2D/3D Pb iodide perovskite crystals.^9^ We did this using a procedure similar to FRAP (fluorescence recovery after photobleaching) using two-photon (2P) confocal microscopy.^4,6^

We used one-photon 1P confocal microscopy to study self-healing at and near single crystal surfaces. In this case, volatile degradation products that form because of photodamage can be lost and, together with reactions with the ambient, make it difficult to isolate the material’s intrinsic self-healing capability.^6^ To prevent these complications, we developed a procedure to encapsulate the films with nonpolar polyisobutylene, PIB, rather than polar polymers (the complete procedure is described in SI Section 1.3, SI Scheme S1). This encapsulation was then used to compare self-healing rates of three HaPs where the A cation was varied (γ-CsPbI_3_, α-FAPbI_3_, and MAPI). We found that the MAPI self-healing rate was considerably smaller than that of the other two HaPs.^16^

Building on that earlier study, we report here on how self-healing of encapsulated, polycrystalline APbI_3_ films is affected by partial substitution of the MA^+^ cation in MAPI by three large (and similar-sized) cations: guanidinium (Gua^+^), acetamidinium (AA^+^), and dimethylammonium (DMA^+^). These three cations differ in the number of hydrogen bonds (H-bonds) with I^–^ (see Table 1).^17−19^ These cations were chosen because reports in the literature showed that substituting them for MA^+^ improves the stability of PV cells (Gua^+^,^19−26^ AA^+^,^22,27,28^ and DMA^+^^29−32^), an effect that is often attributed to reduced halide diffusion.^20,22,33^ Another reason to study mixed A cation HaPs is that in PV cells, HaPs with triple cation compositions are preferred, also because of improved stability, at times attributed to increased configurational entropic stabilization. We chose the least stable pure Pb iodide perovskite to have the highest chance to see effects. Future studies will systematically explore the (MA,FA,Cs)Pb(Br,I)_3_ compositions.

**Table 1:**
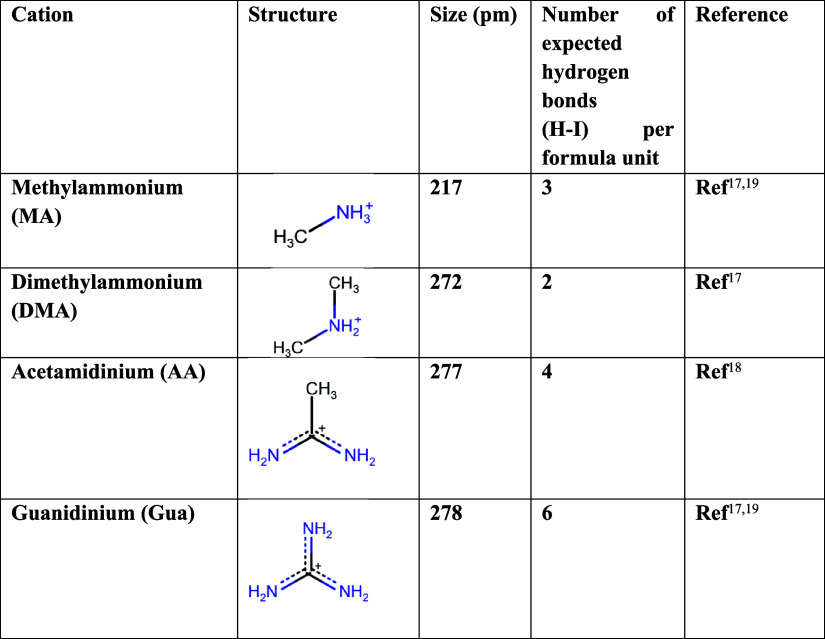
Chemical Structures and Sizes and H-Bonding Possibilities with Various A Cations in APbI_3_

Computational studies showed that the enthalpies of formation of Pb iodide perovskites become more negative, if MA^+^ is substituted for by different cations.^34^ The greater thermodynamic stability of Gua^+^-substituted MAPI was ascribed to two factors: local distortion of the perovskite cages due to the size mismatch (similarly to FA^+^ or Cs^+^ substitution of MA^+^) and differences in possible H-bonding. Tan et al. argued, on the basis of DFT calculations, that the improvement in PV cell stability upon addition of AA was due to reduced halide diffusion as a result of steric impediment and that H-bonding effects do not play a major role.^22^

Experimentally, the origin of the positive effect of the large cations on the solar cell stability remains elusive. Because of the “survival bias” of publications, virtually all articles show an improvement, and not a decrease, of any desired properties upon modification of typical synthesis procedures. This is a problem for building a solid knowledge basis for device improvements that might otherwise not be possible. The results that we present show clear optimal and suboptimal effects that these ions can have on the chemistry, stability, and efficiency of the perovskites. Similar to our earlier study,^6^ we used 1P confocal microscopy where photodamage was created using illumination through a programmed mask (Figure S1).

## 2 Methods

The synthesis of the partially substituted MAPbI_3_ films is reported in detail in the Supporting Information (Section SI 1). We fabricated polycrystalline thin films of pure MAPI and MAPI with 20, 15, and 10 at. % Gua^+^ and 15 or 10 at. % AA^+^ and DMA^+^, by simply substituting the same percentage of MA^+^ in the deposition solution. We did not verify the composition of the resulting thin films but assume that the composition corresponds to that of the solution, as was done in earlier studies.^19,22,27^ Gua (20%) and 15% AA and DMA were close to the upper limits of single-phase films (see XRD patterns in Figure S2a); therefore, we did not use higher concentrations of AA and DMA. We prepared 450–500 nm-thick films by spin-coating (details in SI 1.2) on microscope coverslips (1.5H), to get samples with the typical HaP thickness used in solar cells.

For photodamage and self-healing kinetic measurement, films were encapsulated as described in SI 1.3, while for strain, AFM, and IR spectroscopy measurements, we fabricated unencapsulated films. All experiments were performed with freshly prepared samples.

The main issue with using direct 1P excitation is the high photodamage potential at and near the surfaces, the grains that make up the top of the films. The reason is that volatile degradation products that form because of photodamage (organic or inorganic molecules, such as halogens, organic halides, and further decomposition products of the latter) can be lost. Such a loss will then, by the law of mass action, allow further decomposition. In parallel or as a result of such a loss, surface reactions with the ambient or with contact layers can occur. To prevent material loss and reactions with ambient, we encapsulate the films. Widely used encapsulants with polar groups, such as PMMA, PVA, and PEG, have a tendency to react with photodegradation products such as alkyl amines (e.g., carbonyl of PMMA and PVA reacts with amines). Thus, we developed an encapsulation procedure, using nonpolar polyisobutylene, PIB, which also has a very low (10^–2^–10^–3^ gm/m^2^/day) water vapor permeation rate. To further ensure O_2_ impermeability, glass coverslips were placed on top of the PIB-coated films followed by edge-sealing of the substrate and cover glass with epoxy resin (see SI Section 1.3, SI Scheme S1).

## 3 Results and Discussion

### 3.1 XRD 2θ Peak Shifts, Strain, and H-Bonding in the Films

The Gua-substituted MAPI shows a gradual decrease in the 2θ peak values for 10 and 15% Gua (i.e., a gradual expansion in the lattice parameter as expected when substituting large Gua for MA; Figure S2d). The 20% Gua shows only a small shift compared to the 15% Gua but does show peak broadening, indicative of a range of substitutions from ∼15% up. Both DMA and AA show a clear peak shift from pure MAPI to 10% DMA (and AA) but no apparent further shift for 15% of the substituent (Figure S2b (DMA) and Figure S2c (AA)). However, for DMA, a small shift at higher angles is seen (Figure S3b), which fits with small changes in lattice parameters that are more clearly seen at higher angles. A likely reason (discussed in the SI, p10) is the presence of either a monolayer of APbI_3_ (where A is the large A cation) on the crystals in the films or/and very small clusters of APbI_3_ that do not contribute to the coherent diffraction.

**Figure 1:**
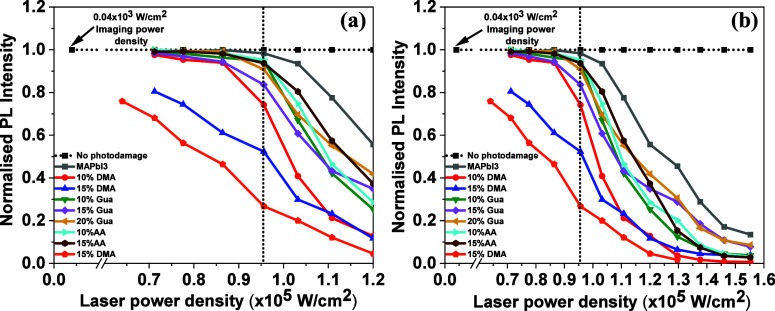
Comparison of photodamage thresholds over narrow (a) and wider (b) laser power ranges in encapsulated MAPbI_3_ thin films, with samples in which some MA^+^ fraction is substituted for by DMA^+^, AA^+^, and Gua^+^, as a function of a 488 nm laser power density of illumination. For all samples, the same 4 × 10^3^ W/cm^2^ power density was used for scanning. The power density to cause photodamage varied from 0.7 to 1.55 × 10^5^ W/cm^2^ (damage inferred from changes in PL intensity). The horizontal dotted line gives the normalized intensities of a nondamaged reference spot. The dotted vertical line is a guide to the eyes that allows comparison of damage thresholds among different compositions at 0.95 × 10^5^ W/cm^2^.

We used out-of-plane X-ray diffraction to assess the presence of strain in the thin films. To that end, we derived Williamson–Hall (W–H) plots from the fwhm of X-ray diffraction peaks (Figures S2–S4 and Table S2). Specifically, we estimated strain values for the various APbI_3_ compositions and found the following order of decreasing anisotropic strain: 15% Gua (0.056%) ∼ 20% Gua (0.052%) ≫ 15% DMA (0.023%) > 10% DMA (0.018%) > 10% Gua (0.008%) > MAPbI_3_ (0.00%). We do not include strain measurements of AA^+^ due to the large variations in the results. We can compare the Gua results with those of (a) Jodlowski et al.^19^ who used a larger range of Gua-MAPI compositions than we did and found essentially a linear relation between the Gua content and strain (Figure 1 in their SI) and (b) Serafini et al.^26^ who found a shift from compressive strain in the pure MAPI to decreasing strain up to ∼7% Gua followed by little further change (up to 15% Gua). Note that in all three cases (refs (18) and (25) and this paper), the strain upon introduction of Gua was positive (tensile). As we shall see later, the strain values measured by us correlate qualitatively with the self-healing kinetics for Gua substitution, but not with the photodamage thresholds. A caveat about the 10% and 15% DMA values is that they should not be directly compared to the other values because these compositions underwent a structural phase transition from tetragonal to cubic, which could have decreased some strain in the original phase (detailed in Figure S3a,b).

To obtain a measure of H-bonding, we preferred polarization-modulation infrared reflection–absorption spectroscopy (PMIRRAS) over conventional ATR (attenuated total reflectance)-IR spectroscopy, as the former technique strongly reduces signals from vibrational modes of H_2_O and CO_2_ present on the sample surface. This allows studying of the −NH stretching vibrations present in hybrid perovskite, with minimal interference from the broad and strong stretching vibrations of H_2_O in the same spectral region. By comparing peak areas of the —NH bands, we find an increase in H-bonding (change in the N–H band frequency) in Gua- and a decrease in DMA-substituted perovskite thin films compared with MAPbI_3_. A detailed description of the results appears in the SI (Figure S5 and Tables S3 and S4).

### 3.2 Photodamage: The Effect of the Laser Power Density

Before describing the results, we give a brief description of the technique that we used to cause the photodamage; detailed information is given in SI Section 4.

**Figure 2:**
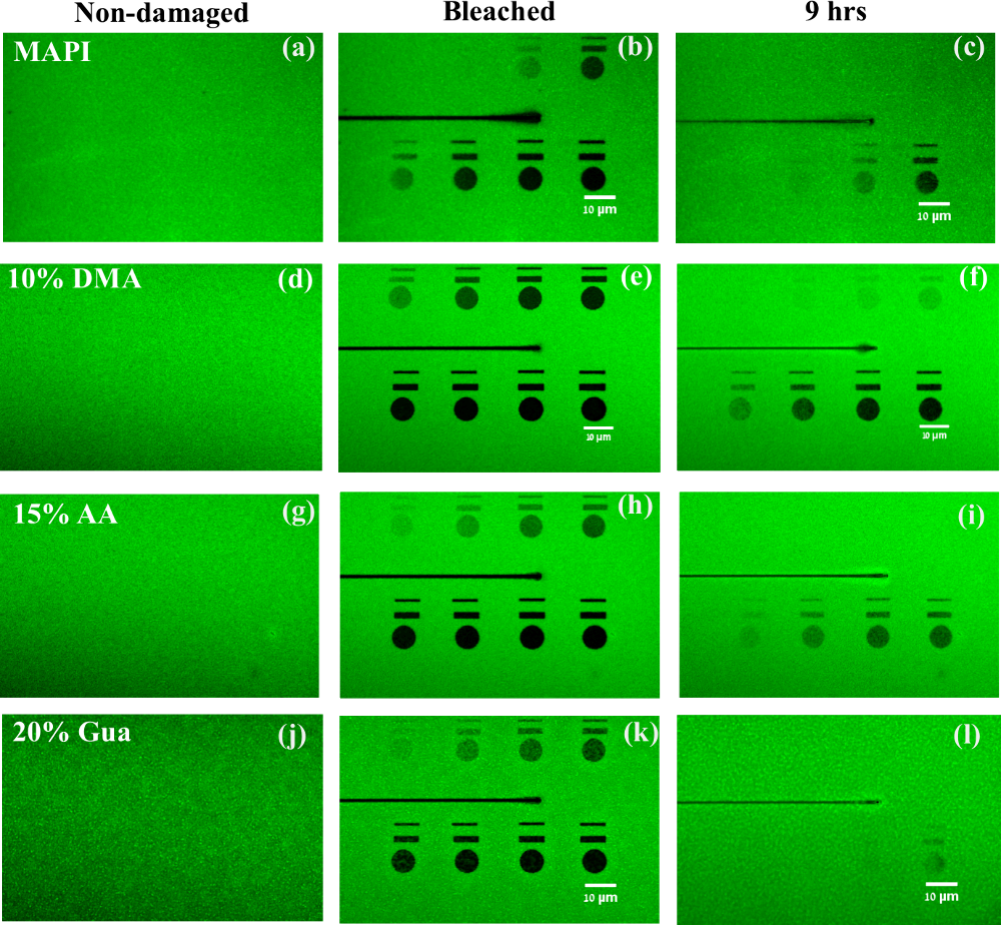
PL images of encapsulated thin films of MAPI, 10% DMA^+^, 15% AA, and 20% Gua^+^-substituted MAPI. The three images in the first column show confocal images of the films; (a,d,g,j) before photodamage, i.e., at *t* < 0. The middle column contains images (b,e,h,k) just after photodamage, i.e., at *t* = 0. The incident laser power density increases from left to right and from top to bottom. The last column (c,f,i,l) shows the PL images of the healed surfaces after 9 h for MAPI (c), 10% DMA^+^ (f), 15% AA (i), and 20% Gua^+^ (l). The black horizontal lines shown in parts b–i are due to an artifact in the programmed pattern used for damaging but, as can be seen, did not influence the measurements of the ROIs.

(a) The sample is excited with supra-band gap 488 nm (2.25 eV) laser light to attain an optimized power density (∼4 × 10^3^ mW/cm^2^) for imaging. Details on the laser intensities, pixel size, and other optical parameters are reported in SI 4.
(b) Photodamage is caused to regions of interest, ROIs, using a mask shown in Figure S1 (see also Figure 2 middle column; cf. Section 3.3), where specific pixels are exposed to nearly 2 orders of magnitude higher laser power density (8 × 10^4^–1.55 × 10^5^ W/cm^2^, high LP) than that used for photoluminescence (PL) excitation. Such high intensity exposure presumably creates light-induced defects that facilitate nonradiative recombination, resulting in a decrease in PL intensity.
(c) Recovery from photodamage with time of each ROI is recorded as a change in the PL intensity, using the same (lower) laser power density as in the first imaging step.

Figure 1 shows the dependence of the PL intensity of the ROIs in encapsulated polycrystalline films, substituted with different cations, immediately after exposure to each of a series of increasing laser power densities. What is plotted are the PL intensities normalized to the PL before damage. More photodamage plots are shown in Figure S6 to show the reproducibility of the data.

As shown in Figure 1a, MAPI is the most resistant to laser damage, requiring power densities >1.0 × 10^5^ W/cm^2^ to cause noticeable damage. We additionally checked the consistency of the extent of photodamage as a function of laser power densities by measuring several spots on the same sample and measuring different samples of the same composition. The variation in the extent of photodamage at a specific power density is very narrow for all the compositions (Figure S6). The substituted samples, excluding DMA ones, had damage onsets varying between and 0.95 × 10^5^ and 0.85 × 10^5^ W/cm^2^ and with little overall difference between them. All the AA and Gua-substituted films start to show damage around 0.90–0.95 × 10^4^ W/cm^2^ with the exception of 15% Gua, which shows damage already at 0.8 × 10^4^ W/cm^2^ (more noticeable in Figure S6e).

The 15% DMA sample behaved very differently with appreciable damage (∼30%) already at the lowest intensity measured, <0.7 × 10^5^ W/cm^2^. The 10% DMA sample showed signs of higher damage at low intensities than all of the non-DMA ones and converged to behave similarly to the 15% DMA sample at high intensities. We also note another unique behavior of the 15% DMA sample (Figure S7), namely, a decrease in PL intensity in the area next to a damaged spot (Figure S7b). The damage in the peripheral area starts to heal within a minute (Figure S7c) and heals completely within 30 min after photodamage (Figure S7d). The peripheral damage may be connected with the high susceptibility to damage of this composition, as noted above (see Figure 1).

These results are consistent with the observation that strain (due to the bulkier cations) makes the material more prone to damage. Still, strain by itself cannot explain the damage observed in Figure 1a. The effect of strain can be offset by differences in H-bonding (Gua > AA > DMA; see Table 1), which can qualitatively explain the data in Figure 1. However, the effects of strain must outweigh those of H-bonding, as the photodamage threshold for strained compositions is lower than that for the MAPbI_3_ films, ignoring effects other than strain and H-bonding.

Note that the power density near the threshold is ∼6 orders of magnitude higher than that of AM1.5 solar irradiation, but the exposure is only for 7 μs/pixel; so, naturally, any instantaneous heating and high carrier density effects will be much more severe than those for solar radiation. As we showed previously using AFM on MAPI,^16^ etch pits occur in the films at intensities that cause >90% PL loss, while no such pits are observed at lower intensities (<1.45 × 10^5^ W/cm^2^, cf. Table S1). Since the entire range of light intensities from slight damage to almost complete damage is only a factor of 2 (from 0.8 to 1.6 × 10^5^ W/cm^2^), it is likely that also at light intensities resulting in the lower damage levels, some material displacement occurs, which is not measurable by AFM. However, the sample architecture (with encapsulation) in healing kinetic studies is entirely different from that used for AFM. Thus, while we cannot exclude that the same effect as seen in AFM occurs here, because of the glass substrate, we cannot assess it (Figure S7 in ref (34)). Also, no damage products can escape; they will remain at the damage site and can undergo matrix reformation over time.

### 3.3 Self-Healing Kinetics: PL Recovery

To compare the healing kinetics of the different films, we collected PL signals from various photodamaged ROIs, obtained using a mask (Figure S1) where each ROI is illuminated with a specific excitation power that increases from left to right and top to bottom and appears as dark circular and rectangular areas in Figure 2 and Figure S1. In Figure 2, the first (Figure 2a–c), second (Figure 2d–f), third (Figure 2g–i), and fourth rows (Figure 2j–l) of the images show self-healing results on MAPI, 10% DMA^+^, 15%AA^+^, and 20% Gua^+^, respectively. Here the first, second, and third columns represent the PL images of the films before damage, just after photodamage, and the recovered state, respectively.

**Figure 3:**
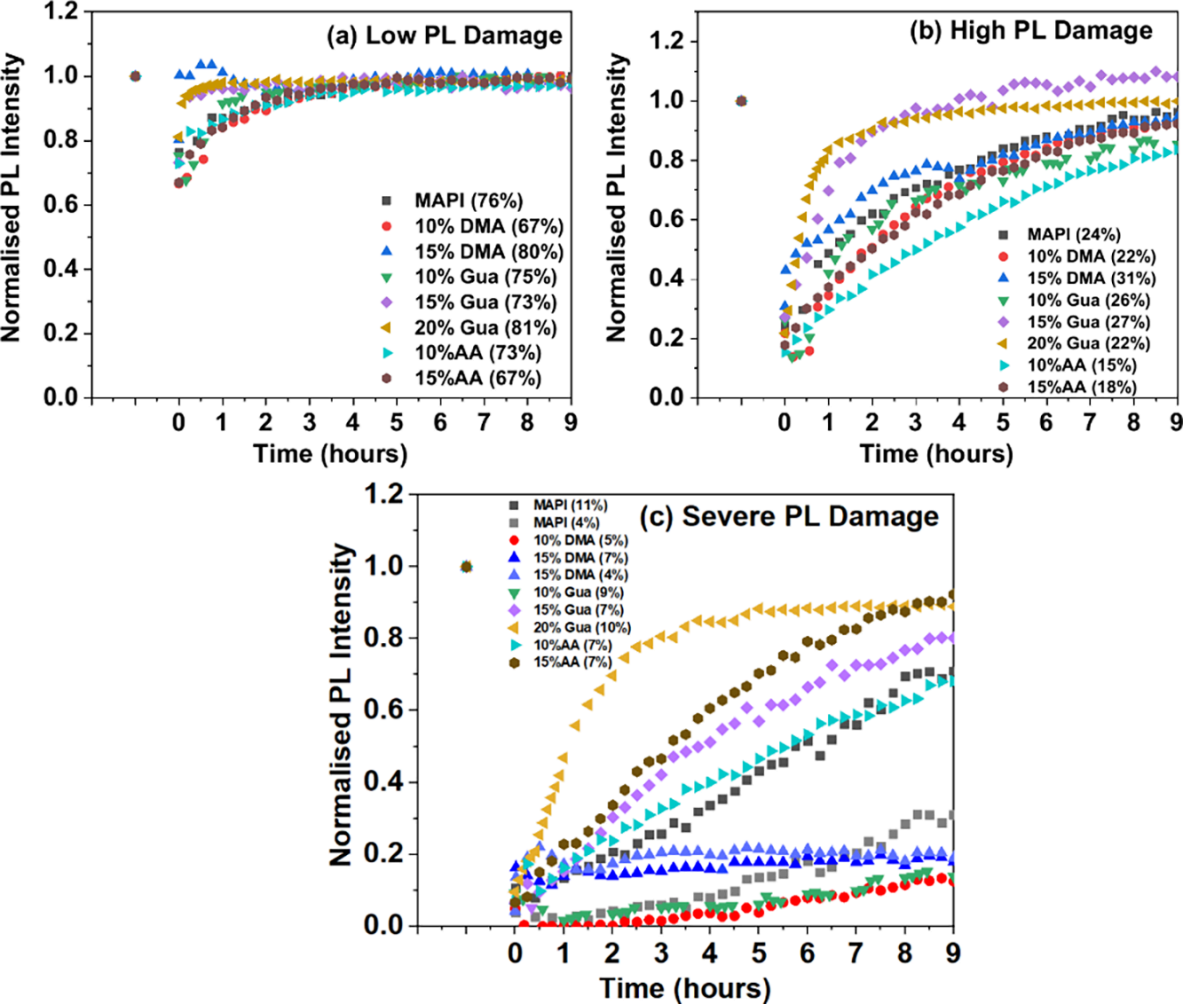
Time-dependent PL recovery for photodamaged ROIs for each of the eight types of films for three different extents of photodamage, as indicated in the figure legends. The extent of damage (the first PL data point measured after damage) is given as % of the undamaged PL after the marker identification symbols as it is often difficult to see this point on the graphs. PL intensities were normalized to that of an adjacent, undamaged area. Each data point shows the average PL intensity of a given ROI at the time, indicated on the *x*-axis. All compositions show broad PL emission in the 720–820 nm range with ∼760 nm emission maximum (with marginal change in the band gap, ranging between 1.55 and 1.58 eV, corresponding to 785–800 nm). PL signals were collected at wavelengths >700 nm using a 700 nm long-pass filter. The time difference between *t* < 0 and *t* = 0 is about 3 s., i.e., it took ∼3 s after photodamage to record the first imaging PL (@ 4 × 10^3^ W/cm^2^); after that, healing was recorded at intervals of 15 min.

For comparison of self-healing kinetics among different compositions, we chose ROIs where the amount of photodamage was similar, rather than exposure to similar LP density (low damage ∼25%, high damage ∼75%, and severely damaged >90% loss of the initial PL intensity). The reason is the earlier analysis that points to the extent of damage as the more relevant parameter than LP density. Figure 3 shows a comparison of the self-healing kinetics among different substituted materials for three different degrees of damage, using agglomerated data from selected circular ROIs in Figure 2. The results are shown as plots of normalized PL intensity vs time. Further, self-healing kinetic plots are shown in SI, Figure S8.

We recorded the time dependence of healing in the dark over 9 h, roughly equivalent to the dark period in a 1 day/night cycle for a solar cell under working conditions. To check if the healed film’s PL is similar to that of the film before damage, PL emission spectra were taken before and after photodamage and then during the time of PL recovery (Figure S9). For all compositions that we studied, no change in the emission spectra was found; all showed λ_max_ ∼760–765 nm.

After low damage (Figure 3a), 15% and 20% Gua and 15% DMA show the fastest self-healing, with all the other compositions exhibiting similar slower rates. Note that for 15% DMA for only slightly more damage than in Figure 3a (see SI, Figure S8a), the self-healing rate is already among the slowest in that group. For the high damage (Figure 3b), 15% and 20% Gua samples show the fastest self-healing, while all the other compositions recover slower and are comparable to each other.

For the severe damage (Figure 3c), 20% Gua is clearly the fastest followed by 15% Gua and 15% AA (similar to each other), and these are closely followed by 10% AA. DMA (15%) behaves anomalously under severe damage: after an initial small extent of self-healing (∼10%), the PL levels off with no further self-healing (two different 15% DMA curves are given to show this effect). Some of the 15% DMA experiments show a gradual PL intensity increase with time although invariably much slower than that for other compositions, as can be seen in Figure S8c for severe damage. The same is very evident also for high damage in Figure S8b and nearly similar damage shown in Figure S8d. Gua (10%) also undergoes anomalously slow self-healing (Figure 3c and Figure S8c).

Within the range of each of the three different extents of damage, there is a moderate range of different damages. The self-healing time is clearly dependent on the degree of damage, and this dependence is particularly apparent for the severe damage cases (compare MAPI at 10.6 and 3.7% PL in Figure 3c, which shows the high and low limits of damage for the same material in the severe damage plots). Thus, we also qualitatively take into account the relatively large differences in damage within any one damage plot. For example, in Figure 3b (high damage), 10% AA with the highest damage (15.4%) and 15% DMA with the lowest damage (30.8%) can therefore be grouped with the other compositions and distinct from 15 and 20% Gua, despite their curves being the high (15% DMA) and low (10% AA) end of their group. After severe damage to 20% Gua, self-healing usually plateaus at ∼90%, i.e., there is no complete self-healing under these conditions. Under low/medium levels of damage, as are more likely to occur in actual devices, the self-healing is complete.

The salient result of the present study, seen most clearly from Figure 3 (also from Figure S8b–d), is that substituting Gua (15 and 20%) for MA in MAPI increases the rate of self-healing substantially, and this difference is greatest at the highest extents of damage. This is the case over the different degrees of damage. The recovery of the 15% AA sample is comparable to that of the 15% Gua sample at severe degrees of damage (Figure 3c). We also note the beneficial effect of 15% DMA at low damage levels but a detrimental effect at severe damage. Smaller fractions (10%) of Gua, AA, or DMA are not sufficient to generate a significant effect on the overall self-healing kinetics.

We also checked the behavior under repeated damage with 20% Gua. To that end, we selected a few ROIs, which were damaged to the same extent (remaining PL: 75, 50, or 25%) and recorded their healing kinetics, followed by several sequential damage events on the same spot, with the same recovery time between subsequent damage events. Our observations show reversibility from one cycle to another (Figure S10).

### 3.4 Cause of Variation in PL Recovery Kinetics with Composition

We now interpret our experimental results and attempt to deduce a mechanism for the observed self-healing observed. The negative free energies of formation of the stable lead halide perovskites are typically very small, a result of yet smaller (or even positive) enthalpies. The numerically comparable (and for stable compounds larger) −*T*Δ*S* term means that the thermodynamic stability of the perovskites (at room temperature) is usually dominated by the entropy term,^35−38^ leading to the so-called entropic stabilization. The small (negative) free energies of the perovskites mean that, based on thermodynamic considerations, decomposition and reformation of the original perovskite are expected to be facile. The easy reformation is attested to by the simple mechanochemical synthesis of these perovskites, usually at room temperature.^39−41^

We noted in Section 3.2 that photodamage is probably associated with ion displacement from the lattice site, where at very high illumination levels, a bulk loss of material can occur, as was detected by AFM (in unencapsulated films). The relatively high ionic mobility in the HaPs (see ref (42) and references therein) can account for self-healing where local ion displacement has occurred as (part of) the damage, and even for more extensive damage (e.g., crack healing of MAPI and FAPI thin films^8^ and self-healing of fractured cm-sized MAPI crystals^7^). Depending on the details of the damage, reformation of the HaP may or may not be an issue, but the possibility of lattice reformation by a thermodynamic driving force (made possible because of small activation energies)^36^ provides more potential pathways for self-healing.

Based on the calculated thermodynamic stabilization of MAPI by substitution with various A-site cations,^34^ this same driving force might rationalize that all the substituted compounds recover more readily from photodamage than pure MAPI. However, while true for Gua and AA, it leaves open the question of why DMA does not (except at low damage levels).

We note that after completion of the spin-coating step during film preparation (before annealing), MAPI turns light brown, AA and Gua dark brown, while DMA-substituted ones are pale yellow (Figure S11). These visual results suggest that formation of photoactive perovskite DMA-substituted MAPI is more difficult than that of all the other samples at room temperature. Additionally, the prominent darkening of the 15% AA and 20% Gua films compared to MAPI suggests that the former two form more readily than pure MAPI; if so, then that can be a factor for faster self-healing in the AA and Gua-substituted MAPI films than in the other ones. Indirect support for this idea comes from the report that Gua doping of the (thermodynamically unstable) black phase of CsPbI_2_Br lowers the annealing temperature needed to form that phase, by way of more facile crystallization and stabilization.^43^

We now consider the kinetics of self-healing. Assuming that some form of ion migration is necessary for self-healing, we can compare self-healing rates to migration rates of the different ionic species that are thought to be present and mobile. The first question is if species move over long (at least hundreds of nm) or short (<100 nm) distances, on average. Based on Figure 2, we see that the damaged area is several orders of magnitude larger than the atomic scale. The time lapse video shows uniform recovery of PL over the damaged area (with no sign of self-healing initiated from the perimeters of the ROIs inward). Therefore, we can assume that ion diffusion is responsible for the healing and that it occurs on a scale that is significantly smaller than the μm-s size of the ROIs. Another possibly more convincing reason to believe that the relevant ion diffusion needed for self-healing is short-range is intuitive. Displacement of the ions in the rate-determining step (rds) for self-healing will occur over the whole area of the sample exposed to the damaging laser beam. Therefore, even if a large displacement of the relevant ions were to occur, since there is no expected directionality in the damage (except maybe at the boundary of the ROI, and we do not see even this as noted previously), on average, there would be a displaced ion not far from a related vacancy. Taking an (RT) iodide diffusion coefficient for MAPI, *D*_I_- = 10^–12^–10^–14^ cm^2^/s^42^ gives a diffusion length, *L*, ∼500–50 nm after 1.5 h (a time during which roughly half of self-healing has occurred on average for low and high PL damage [Figure 3]). However, if the rate-limiting ion diffusion has the submicrometer scale that we argued for above, we would expect complete self-healing well within that 1.5 h. So, it is unlikely that I^–^ diffusion is the rds in the self-healing process for MAPI. However, Ferdani et al.^34^ found from a combined experimental–theoretical study that just 5% Gua substitution reduces *D* by 5 orders of magnitude. If true and *D*_I_- ≈ 10^–17^ cm^2^/s (→ L_I_- ≈ 2.5 nm in 1.5 h) and even if the reduction is not 5 orders of magnitude, but two or three orders, then it would seem that we should not rule out I^–^ diffusion as the rds in all the substituted MAPI compounds in this study. However, since we find that 10% Gua has no appreciable effect on the self-healing, this again would imply that I^–^ diffusion is not the rds as if it were, we would expect some effect from 10% Gua.

Slower diffusing A^+^ and Pb^2+^ cations may also be caused by the damage. We could only find upper limits for *D* for both cations (which was essentially the same for both cations) in MAPI: 6 × 10^–13^ and 4 × 10^–15^ cm^2^/s at 378 and 343 K, respectively,^44^ which can be extrapolated to ∼2 × 10^–17^ cm^2^/s at RT. Therefore, for these two cations, *L* is ∼3 nm after 1.5 h. The rds diffusion of these cations would, therefore, be a better match for the observed self-healing rates, assuming that the cations were only displaced by a small distance by the damage. Incidentally, while we could not find any other value of *D* for Pb^2+^ in HaPs, we could extrapolate an RT value for Pb^2+^ in PbBr_2_ from a radioactive isotope tracer study, which gave a value between 10^–15^ and 10^–16^ cm^2^/s.^45^ We should note that values of the activation energies, *E*_a_, for defect-mediated ion migration for MA^+^ and Pb^2+^ have been calculated by several groups and summarized in ref (46). While these values vary considerably among themselves, the *E*_a_ of the Pb^2+^ is considerably greater than that of MA^+^, suggesting a higher *D* for the latter.

We next consider the effects of other parameters on self-healing rates that we observed to see whether any convincing correlation exists.

Strain, due to the presence of the large A cations in the MAPI, may affect self-healing. As noted, we measured strain in the films from the XRD data (Table S2). The highest strain is for 15 and 20% Gua, intermediate for 10% DMA and 15% DMA (complicated by a tetragonal to cubic phase change), very small strain for 10% Gua, and no strain for MAPI. We also note the results of previous studies mentioned earlier that, for increasing Gua substitution of MA, tensile strain was found to increase,^19,26^ linear up to ∼18 at. % Gua,^19^ or, starting with compressive strain at 0%, becoming strain-free at ∼7 at. % and remaining such until 15 at. %, the highest Gua substitution measured.^26^ The fact that the two fastest healing samples are also the ones with the highest strain may suggest a link between strain and self-healing kinetics. Unfortunately, results of AA strain measurements were insufficiently reproducible in our study; however, considering Figure S9 from the report of Tan et al.^22^ where nearly identical lattice volume expansions were measured, when AA and Gua were substituted for MA in MAPI, we can expect little difference in strain in films where MA is substituted by these cations. Overall, we conclude that there seems to be a link between strain and self-healing in these samples. We recall that 10% Gua undergoes anomalously slow self-healing after severe damage. Since 15% Gua and more so 20% Gua self-healing are faster than MAPI, this suggests that there are two effects of the Gua substitution on self-healing: one decreasing the rate of self-healing at (relatively) low concentrations of Gua and the other effect increasing the rate at higher concentrations. Since 10% Gua introduced very little strain into the films, it is likely that this small amount of strain is not the cause of the slow self-healing of Gua after severe damage, but it might well be for the more highly strained 15 and 20% Gua films.

H-bonding will vary considerably in differently substituted MAPI compounds. Some reports attribute the additional thermodynamic stability with Gua, compared to pure MAPI to the greater number of H-bonds, 6 H–X bonds/substituted formula unit, in the former, as MAPI has 3 H–X bonds/formula unit, AA has 4, and DMA has 2 (cf. Table 1).^19,43^ The effect of H-bonds on thermodynamic stabilization is well-known and well-documented for biomolecules, including DNA,^47^ as well as for pure water.^48^ In our work, there is in general a positive correlation between the number of H-bonds and the self-healing rate. However, in a recent comparison of self-healing rates between encapsulated polycrystalline thin films of MAPI with perovskite-structured FAPI and CsPI ones, CsPI (with no H-bonding) and FAPI (4 H-bonds) healed at a similar rate and much faster than MAPI (3 H-bonds).^16^ The report of Svane et al.^17^ notes that MA^+^ tends to form stronger hydrogen bonds than FA^+^ due to a higher dipole moment (μ_A_ = 2.29 D vs 0.21 D): AA and Gua with μ_A_ 0.21 and 0 D, respectively, could be expected to behave similarly to FA^+^. If so, then the strength of individual H-bonds might play a role in the higher MAPI damage than that of the others because, as shown in refs^17^ and (19), H-bonding strength involves more than just the number of bonds. Still, the total lack of any correlation between number of bonds and the self-healing rate in our previous work suggests that H-bonding may not be a major issue for self-healing kinetics in HaPs in general, which would be in contrast to self-healing in organic (bio)polymers.^49^

Comparing self-healing rates with dipole moments of the various A cations (Table 2) shows an anticorrelation. The two cations with relatively high dipole moments (MA and DMA with μ_A_ = 2.29 and 1.49 D, respectively) show the slowest self-healing, while AA and Gua with smaller or zero dipole moments show faster self-healing. Pazoki et al.^50^ using DFT studies of tetragonal phases of MAPI, FAPI, and CsPI deduced that a stronger A^+^ dipole facilitates I^–^ vacancy creation but impedes I^–^ vacancy migration. However, another theoretical study, using dynamic simulations, predicted an increase in the rate of an I^–^ vacancy migration for a stronger A^+^ dipole.^46^ Thus, at this stage, it seems possible that the A^+^ dipole moment affects self-healing, but likely, it has effects on other properties, relevant to self-healing as well, preventing any clear prediction. Also, it should be remembered that the samples in this study are still mainly MA with up to a maximum of 20% substituted A cation (20% Gua), so that any dipole moment effect will be limited.

**Table 2:** Comparisons of Self-Healing Kinetics among Various Organic Cation-Substituted Films Considered in This Study and FAPI and CsPI^16^ Based on Various Properties of the A Cation

**composition**	**size of the A cation [pm]**	**strain**	**dipole moment of the A**^**+**^**cation [Debye]**	*n***H–I bonds/substituted formula unit**	**extent of healing from 95 to 100% PL damage (healing time)**
MAPbI_3_	217	unstrained (T)	2.29	3H-bonds	>15% healing (9 h)
10% DMA	277 (DMA)	T to C	1.46–1.8	2H-bonds	>15% healing (9 h)
15% DMA		T to C			>30% healing (9 h)
10% AA			0.21	4H-bonds	>12% healing (9 h)
15% AA					>60% healing (9 h)
10% Gua	278 (Gua)	slight strain (T)	0	6H-bonds	>10% healing (9 h)
15% Gua		strained (T)	0		>80% healing (9 h)
20% Gua		strained (T)	0		>90% healing (6 h)
FAPbI_3_ (black)	257 (FA)	strained	0.21	4H-bonds	100% healing (1.5 h)
CsPbI_3_ (black)	167 (Cs)	strained	spherical	no H-bonds	>100% healing (1.5 h); brightening

Making the reasonable assumption that some short-range ion diffusion is required for self-healing, the rate-determining diffusion is probably of either MA^+^ or Pb^2+^ for MAPI. It could be any of the cations for the substituted MAPI samples, although it is reasonable to assume that the large A cations will diffuse slower than MA^+^. The question then is what affects the rate of diffusion of the defects, i.e., the ions in the damaged volume? The strength of the H-bonding would be expected to affect this for MA^+^. We find a positive correlation between the number of H-bonds (which we use here as a proxy for strength of the bonds in the absence of other information) and the self-healing rate. However, we would expect a negative correlation (weaker bonding leading to more likely diffusion of the MA^+^). It is, though, also possible that the Pb^2+^ diffusion is the rds, in which case the above argument is invalid.

The (tensile) strain in the films due to the large cations may cause many effects, but it is difficult to link any one specific effect to the self-healing kinetics.

Finally, the anticorrelation between self-healing rates and dipole moment of the substituting A^+^ cation can be traced to a number of factors: still there is, at this point, insufficient reliable information on which to base a hypothesis.

## 4 Conclusions

Our findings support the commonly held understanding that the inherent low formation energies and ease of ion migration in halide perovskites facilitate rapid lattice formation and reformation, even at room temperature (RT). Notably, in conventional semiconductors, both of these features are uncommon and generally considered problematic; together, they are unprecedented.

Specifically, in the Pb iodide perovskite compositions examined, substituting guanidinium (Gua) for methylammonium (MA) at high concentrations (≥15%) significantly enhances self-healing kinetics compared with that of pure methylammonium lead iodide. The effects of acetamidinium (AA) substitution are comparable to those of Gua, especially under conditions of severe damage. Based on the timescale of SH, we propose that the rate-limiting step for self-healing is the diffusion of MA^+^ and/or Pb^2+^, rather than that of iodide, the species often assumed to be the fast diffuser in Pb iodide perovskites.

From a broader perspective, the thermodynamics of material (re)formation might also influence these processes, although pinpointing the exact role of varying ion diffusion rates remains challenging. However, certain experimental observations, such as the positive correlation between strain and self-healing rates and the inverse correlation with dipole moments, may well relate to what retards or speeds up defect/ion diffusion. While there is also some (positive) correlation between H-bonding and self-healing, results of other studies suggest that this might not be very important in self-healing.

To arrive at the mechanisms that facilitate or impede SH, future research on quantifying changes in thermodynamic properties related to material formation and degradation following A cation substitution may be helpful. Additionally, operando nuclear magnetic resonance (NMR) studies might help to identify the dynamic processes governing self-healing. Such follow-up studies can point to the factors essential for optimizing the self-healing capabilities of HaPs, possibly allowing control over them and the materials’ resilience, leading to more durable (opto)electronic devices.

## Supporting Information Available

Detailed thin-film fabrication and encapsulation, X-ray diffraction and strain and crystallite size calculation, PMIRRAS measurement, detailed one-photon confocal microscopy: imaging and photobleaching conditions, photodamage as a function of laser power plot and additional self-healing kinetic plots for different degrees of damage for various composition, photoluminescence spectra of nondamaged, damaged, and recovered spots, and repeated photodamage and fast healing events on the same ROI for the 20% Gua composition (PDF)
