# Supplementary material for: Guanidinium Substitution Improves Self-Healing and Photodamage Resilience of MAPbI3
Source: J Phys Chem C Nanomater Interfaces. 2024 Nov 20;128(47):19999–20008. doi: 10.1021/acs.jpcc.4c06090 (PMC11613547; doi:10.1021/acs.jpcc.4c06090)
Supplement: Supplementary file 1 — jp4c06090_si_001.pdf [file jp4c06090_si_001.pdf]

# Guanidinium Substitution Improves Self-Healing and Photodamage Resilience of $MAPbI_3$

Pallavi Singh[1], Davide Raffaele Ceratti[2], Yahel Soffer[3], Sudipta Bera[1], Yishay Feldman[4], Michael Elbaum[5], Dan Oron[1], David Cahen[1]*, Gary Hodes[1]*

[1]Dept. of Molecular Chem. & Materials Science, Weizmann Institute of Science, Rehovot, 7610001, Israel

[2] PSL University, CNRS, Chimie ParisTech, Institut de Recherche de Chimie Paris, Physical Chemistry of Surfaces Group, 11 rue Pierre et Marie Curie, Paris, 75005, France

[3]Dept. of Physics of Complex Systems Weizmann Institute of Science, Rehovot, 7610001, Israel

[4] Dept. of Chemical Research Support, Weizmann Institute of Science, Rehovot, 7610001, Israel

Dept. of Chemical & Biological Physics, Weizmann Institute of Science, Rehovot, 7610001, Israel

* gary.hodes@weizmann.ac.il, david.cahen@weizmann.ac.il

This supplementary information file contains sections for preparation of thin polycrystalline films of the halide perovskites (HaPs), Pb iodide perovskites in our case, their encapsulation procedure and other experimental details of the techniques used in this study. Further, we present various experimental data, which are discussed in the main manuscript, but not elaborated there.

**Experimental details:**

SI 1. Thin film fabrication and encapsulation.
SI 2. X-Ray Diffraction measurement of the perovskite thin film.
SI 3. Polarization Modulated IR reflection absorption spectroscopy (PM-IRRAS) measurement.
SI 4. One-Photon Confocal Microscopy: Imaging and photo-bleaching conditions.

**Supplementary Figures and Tables:**

Scheme S1 Scheme of film encapsulation and geometry of measurement.

Figure S1. Pictorial representation of programmed mask with laser power percentages varying from 1% to 38% used for FRAP study with 1P-confocal microscopy.
Figure S2. Diffraction pattern of the substituted HaP thin films.
Figure S3. Diffractograms showing change from tetragonal to cubic phase in 10% and 15% DMA. Also, diffraction plane obtained from the secondary phase in 20% DMA films.
Figure S4. Strain and crystallite size calculation for HaP thin films using Williamson-Hall (WH) plots.
Figure S5. PMIRRAS spectra of the perovskite thin films substituted with different fractions of $DMA^+$ and $Gua^+$ cations.

Figure S6. Photodamage as a function of laser power: additional plots to supplement Figure 1.
Figure S7. Damage and recovery of surrounding areas (other than regions of interests, ROIs) in 15% DMA HaP films.
Figure S8. Additional self-healing kinetic plots for different degrees of damage.
Figure S9. Photoluminescence spectra of HaP thin films, substituted with different fractions of $DMA^+$ and $Gua^+$ cations (for $MA^+$), before and after photo-damage in the 500-800 nm region.
Figure S10. Repeated photo-damage and fast healing events on the same ROI in 20% Gua HaP films.
Figure S11. Freshly prepared thin films of MAPI and of MAPI with partial MA for DMA, AA and Gua substitution, before thermal annealing.

Table S1. The fraction of laser power, LP, in % of the full LP, in power units and as power density for a laser beam area of 0.25 μm$^2$.
Table S2. Shows compositions and their corresponding strain calculated using W-H plots obtained from XRD patterns; errors are mentioned in the parentheses.
Table S3. Comparison of peak positions of medium to strong intensity —NH stretching and bending vibrations in HaP thin films, substituted with different fractions of $DMA^+$ and $Gua^+$ cations.
Table S4. Comparison of relative peak intensity ratios of $—NH_2$ and $—NH_3^+$ stretching and bending vibrations of HaP thin films, substituted with different fractions of $Gua^+$ cations.

**Experimental section:**

## SI 1. Thin film fabrication and Encapsulation

### SI 1.1. Chemicals:

Lead iodide was purchased from Sigma Aldrich. All organic quaternary ammonium salts such as methylammonium iodide, dimethylammonium iodide and guanidinium iodide were purchased from Greatcell Solar. Solvents anhydrous dimethyl formamide (DMF) and anhy. dimethyl sulfoxide (DMSO) were purchased from Sigma Aldrich. Polyisobutylene polymer was purchased from J&K scientific. All the materials were used as received. Microscope coverslip (number 1.5H) with thickness 175±5 μm was used a substrate and purchased from Marienfeld.

### SI 1.2. Thin film deposition:

We use the microscope coverslip of this specific thickness (170 ± 5 μm thick) as a substrate because of the limitation imposed by the confocal microscope objective, as it was corrected for this specific thickness. Substrates were cleaned by rinsing sequentially in acetone, ethanol and de-ionized water for 10 min. each, followed by UV-ozone treatment to increase hydrophilicity for improved surface coverage. All processes, including solution preparations, the spin coating to deposit thin films and annealing, as well as the encapsulation, were done in a dry $N_2$-filled glove box maintained at ≤ 0.1 ppm $H_2O$ and 0.1 ppm $O_2$. Before deposition, all solutions were stirred at 70 ºC for 2-3 hours and then cooled to RT. All films were spin coated at 4000 rpm, with acceleration 4000 rpm/sec, for 30

sec. 150 μL chlorobenzene antisolvent was introduced 10 sec after the start of spinning. The films were annealed at 65 $^{0}$C for 1 min followed by 100 $^{0}$C for 10 min.

**SI 1.2.1. $MAPbI_3$:** A 1.4 M solution of MAI and $PbI_2$ (1:1 molar ratio) was prepared in DMF : DMSO (9:1). The films turned light brown during the spin-coating (Figure 6), in contrast to what is the case for the DMA-substituted ones (see below).

**SI 1.2.2. Preparation of films where 10% or 15% or 20%Gua** is substituted for $MA^+$: 1.4 M solutions were prepared, using 0.2 or 0.15 or 0.1 : 0.8 or 0.85 or 0.9 : 1 GuaI : MAI : $PbI_2$ molar ratios of precursors in DMF : DMSO (9:1). The films with 15% and 20% Gua turned brown and more reflective/shiny already during spin-coating, and the film with 10% Gua less so (Figure 6).

**SI 1.2.3. Preparation of films where 10% and 15% DMA** is substituted for $MA^+$: 1.4 M solutions were prepared using 0.1 (0.15) : 0.9 (0.95) : 1 DMAI : MAI : $PbI_2$ molar ratios of precursors in DMF : DMSO (9:1). The films became yellow and less shiny during spin-coating, i.e. markedly different from the pure MA and Gua-substituted MA iodide perovskites film types, which are more shiny and light brown ($MAPbI_3$) and dark brown (15% and 20% Gua), already on the spin-coater.

**SI 1.2.4. Preparation of films where 10% and 15% AA:** preparation of AA substituted film is similar to DMA substituted films except acetamidium iodide was been used instead of DMAI. As-deposited AA-substituted films are similar in color to Gua-substituted films.

The significance of change in color and reflectance of the films *before annealing* is highlighted because this might be related with the stability of these perovskite materials, as is discussed in the section "mechanism of self-healing" in the main text.

**SI 1.3. Sample Encapsulation for Self-healing measurement:**

All steps of sample encapsulation were performed inside a dry $N_2$-filled glove box. After deposition and annealing of the perovskite films, the edges of the substrate were cleaned from all directions with a blade as seen in the Scheme S1 (Step 3). $N_2$ gas was passed over the substrate to remove any dust particles from the top surface while edge cleaning.

A 12 mg/mL solution of poly-iso-butylene (PIB) in toluene was prepared. It took 5-6 hrs. to completely dissolve the polymer in the solvent. This solution was then drop-cast on the perovskite film and annealed at 100 ºC for 2-3 min, then cooled and the whole process was repeated once more. To further block any ingression of $H_2O$ and $O_2$, "Devcon 5 min epoxy" was applied as an edge sealant on the cleaned edges, followed by placing another coverslip on the top of the perovskite film. "Devcon 5 min epoxy" has 2 parts, the hardener and the resin, which were used in equal quantity, and mixed well for one minute before applying the product to the edges. The other coverslip was placed gently on the top of the sample. Subsequently the coverslips were pressed gently on the edges so that the edge sealant distributed uniformly at the edges without leaving any air gaps in the edge-sealing layer. The complete assembly was then clipped with a binder/paper clip to ensure tight packing of the two coverslips. After an hour the sample was ready for the FRAP experiments. We find that samples, encapsulated only with the PIB polymer, can degrade in 1-2 weeks, while samples encapsulated as presented in Scheme S1, remain physically unchanged even after several years.

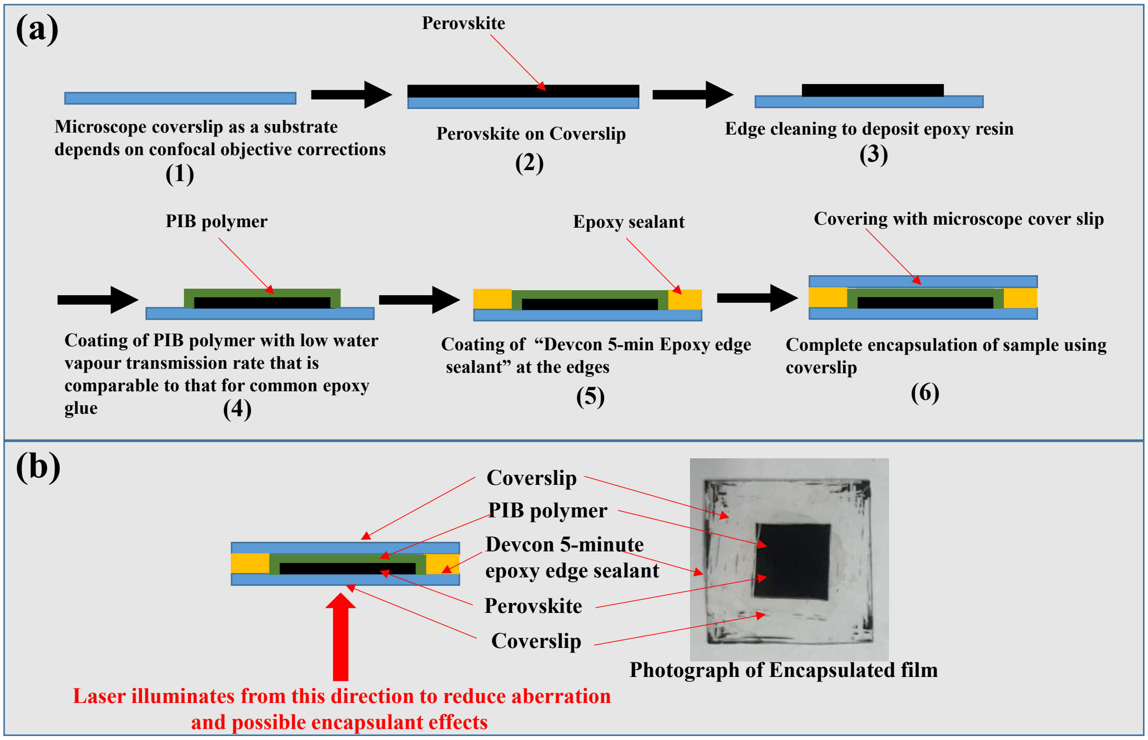


*Scheme S1. (a) Encapsulation scheme of polycrystalline perovskite thin film for self-healing measurements. (b) LEFT: Illumination geometry for self-healing measurement using 1Photon-FRAP; RIGHT: Photograph of encapsulated sample.*

We used PIB to encapsulate the films, because it is a pure aliphatic hydrocarbon, and does not contain any polar group. Polar polymers such as PMMA and polyvinyl acetate (PVA) have a tendency to bind with chemical species produced after iodide perovskite photodamage ($I_2$, MA), which might affect self- healing (for example the amine produced after photo-damage can react with the ester group of PMMA or PVA).

## SI 2. X-Ray Diffraction measurement of the perovskite thin films:

The XRD patterns were recorded at ambient using a Rigaku TTRAX-III X-ray diffractometer equipped with a rotating anode X-ray tube operating at 50kV/200 mA and with a scintillation counter that was aligned to intersect the diffracted beam after it had passed the graphite monochromator to sufficiently reduce $K_\beta$ radiation. The data were acquired at specular conditions over the range $2\theta$ = 5–60° with a scan rate of 1°/min and a step size of 0.02°. Using the Jade Pro software (Materials Data, US), XRD analysis served to check for phase purity, find the presence of secondary phases and assess strain.

**Sample preparation for XRD:** HaP films were deposited on a microscope coverslip (number 1.5H) with thickness 175±5 μm (similar coverslip used for self-healing studies). It is encapsulated once with PIB polymer using spin-coating (PIB solution concentration is 10 mg/mL and spin speed 2000 rpm) to reduce the effect of ambient on the thin films during measurement.

**SI 3. Polarization Modulation Infrared reflection absorption spectroscopy (PM-IRRAS)** measurements were performed to study the vibrational modes of the HaP thin films, with different cations substituted for a fraction of the MA. The system works with a PEM (photoelastic modulation) module, which is integrated in a single-channel Nicolet 6700 spectrometer with a grazing angle accessory and a liquid $N_2$-cooled MCT detector. Dedicated Omnic 8.1 software was used for IRRAS data collection and analysis. For each sample, the PM-IRRAS spectrum was collected over 950 scans at 1 $cm^{-1}$ resolution with an incident angle $80^o$; the resulting spectrum was the averaged result over all the scans.

**Sample preparation for PMIRRAS:** HaP films were deposited on pre-cleaned Au-coated semi-insulating Si substrates (Au/Si) inside the glove box (0.1% $H_2O$ and 0.1 ppm $O_2$) and measurements were conducted in ambient condition. As this technique involves reflection from the substrate surface with grazing incidence, we prepared very thin films using diluted solutions (0.7 M). This technique is preferred over ATR (Attenuated total reflectance)-IR spectroscopy, because it strongly reduces signals due to the vibrational modes from $H_2O$ and $CO_2$ absorption on the sample surface, which makes studying –NH vibrations feasible as broad and strong stretching vibrations for $H_2O$ also appear in the same region.

**SI 4. One-photon confocal microscopy: Imaging and photo-bleaching conditions:**

The methods are described in ref. Singh *et al.*[1], but are repeated here, for the sake of completeness of the experimental details of the work.

1. FRAP studies were carried out on an Olympus Fluoview confocal microscope (BX50WI) equipped with several lasers as excitation sources. A supra-bandgap CW laser beam (488 nm) illuminated the sample surface and the resulting photoluminescence (PL) signal was measured. The laser wavelength was selected as the absorption coefficient at this wavelength is sufficiently high so that most of the absorption occurs in the top 100 nm of the layer. Sufficiently low beam intensity was used to avoid any measurable damage and this defines the "non-damage" baseline PL.
   For effective gathering of the diffused PL from the surface of the polycrystalline films, we used a 60x oil immersion objective with 1.4 numerical aperture. The area of exposed ROIs is the same in all cases, irrespective of power density and material composition.
   The laser was raster-scanned over the sample with a scan rate of 2.71 sec/scan over 512×512 pixels with a dwell time per pixel of 7.2 μs, which is constant for all compositions. For all perovskite compositions we use the same power density of $0.04\times10^5$ W/$cm^2$ for imaging, which is only 1% of total laser power of 488 nm. We used a 700 nm barrier filter to block any 488 nm reflected light. The confocal aperture positioned in front of the detector, which defines the analysis volume of the sample, was 300 $\mu$m in diameter.

2. The sample surface was exposed to 1-2 orders of magnitude higher laser power density than the imaging power to cause localized photo-damage in the regions of interest (ROIs appear as circular/rectangular spots in Figures 2) with the help of a programmed mask (Figure S1). This mask allows the setting of laser power percentages (18%-38% including 1%), and we calculated corresponding power densities, which vary from $0.04\times10^5$ W/$cm^2$ to $1.55\times10^5$ W/$cm^2$ provided in Table S1 with beam area 0.25 μ$m^2$. We found $1.55\times10^5$ W/$cm^2$ suffices to cause 90-100% PL loss in all cases; therefore, this power density was

set as a maximum in the mask. The study involving damage threshold comparison was limited to this step only; however, the study of healing kinetics involved the following additional step.

3. To observe recovery kinetics, which starts just after photo-damage, we track the PL in the ROIs over time after photodamage using low power density used for imaging. Measurements were done in ambient condition on encapsulated samples. The ambient relative humidity, RH, varied from 35 to 40% and the temperature from 24 to 25 ºC.
4. All the above steps were followed by acquisition of the PL spectrum (before and immediately after, as well as during recovery of the photo-damage) to check if the material had changed (decomposition or phase transformation).

In order to plot recovery kinetics data as presented in Fig 3, we considered a PL count of a circular area (5 µm diameter) in the center of the damaged circular spot.

It is important to consider that since the bandgap of all our materials is approximately 1.6 eV, some 0.9 eV of the exciting photon energy, ~2.5 eV is released as heat by carrier thermalization, the effect of which decreases exponentially from incident to bottom surface of the film. The remaining 1.6 eV are rather uniformly distributed throughout the film thickness as the diffusion lengths of the photogenerated carriers are sufficient to have the carriers reach both interfaces (those with the glass onto which the sample is deposited and that with the poly-isobutylene (PIB), that is deposited on top of the film; cf. Scheme S1, above). The result is an almost uniform charge density and, thus, recombination rate (as long as surface recombination is insignificant, compared to that within the grains). Because there is no further diffusion of charges in the Z direction and the illumination time is around 7.2 microseconds, the PL reaches a steady state in this time (see this section).

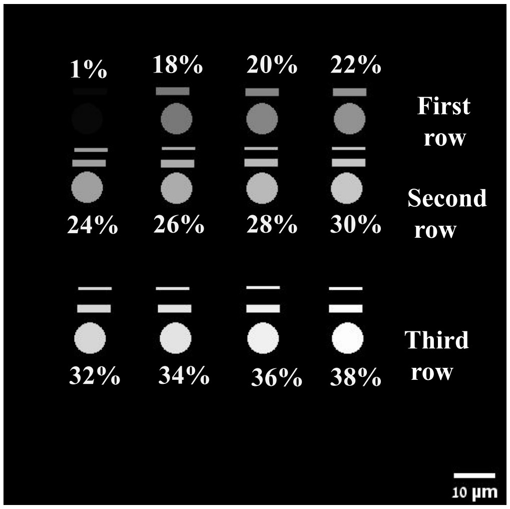


***Figure S1.*** *Pictorial representation of programmed mask with laser power percentages varying from 1% to 38% used for above FRAP study with 1P-confocal microscopy.*

| Laser power [%] | Corresponding laser power [µW] | beam area [µm$^2$] | Power density [$10^5$ W/cm$^2$] |
|---|---|---|---|
| 1 | 10 | 0.25 | 0.04 |
| 14 | 140 | | 0.56 |
| 16 | 160 | | 0.64 |
| 18 | 178 | | 0.71 |
| 20 | 194 | | 0.77 |
| 22 | 216 | | 0.86 |
| 24 | 239 | | 0.95 |
| 26 | 258 | | 1.03 |
| 28 | 277 | | 1.11 |
| 30 | 300 | | 1.20 |
| 32 | 324 | | 1.30 |
| 34 | 344 | | 1.38 |
| 36 | 365 | | 1.46 |
| 38 | 388 | | 1.55 |

***Table S1.*** *The fraction of laser power, LP, in % of the full LP, in power units and as power density for a laser beam diameter of 0.25* $\mu m^2$.

## Supplementary data and interpretation

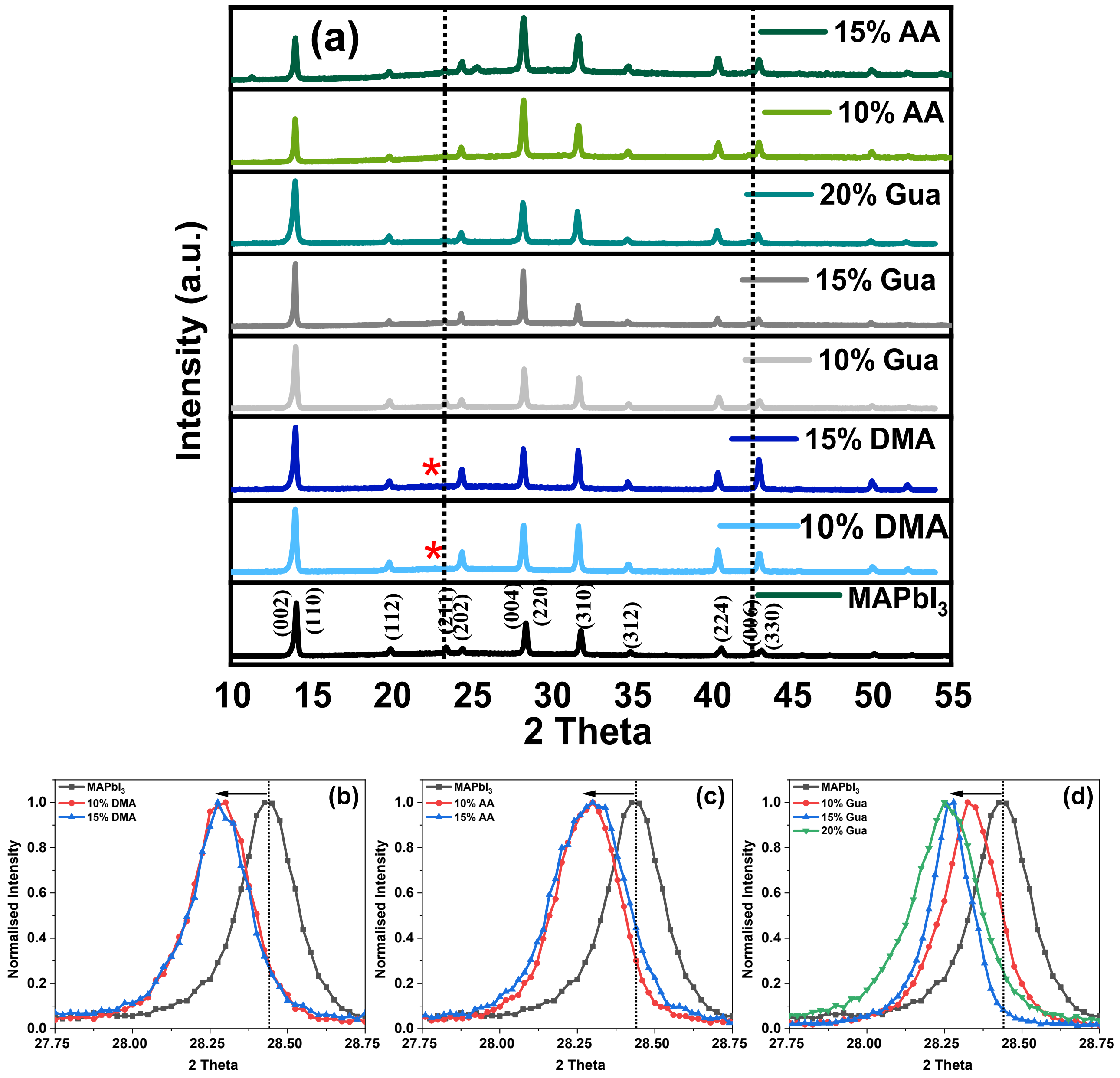


***Figure S2.*** *(**a**) Diffraction pattern of the Pb iodide perovskite thin films with different fractions, of $MA^+$ substituted by $DMA^+$ $AA^+$ or $Gua^+$; The reflections of $MAPbI_3$ (room temperature, tetragonal phase) are labelled (dotted black line) with * in the other patterns corresponding to the absence of a peak. (b-d) Shift towards lower 2-theta show increased interplanar distance in perovskite lattice on substitution of various bulky cations.*

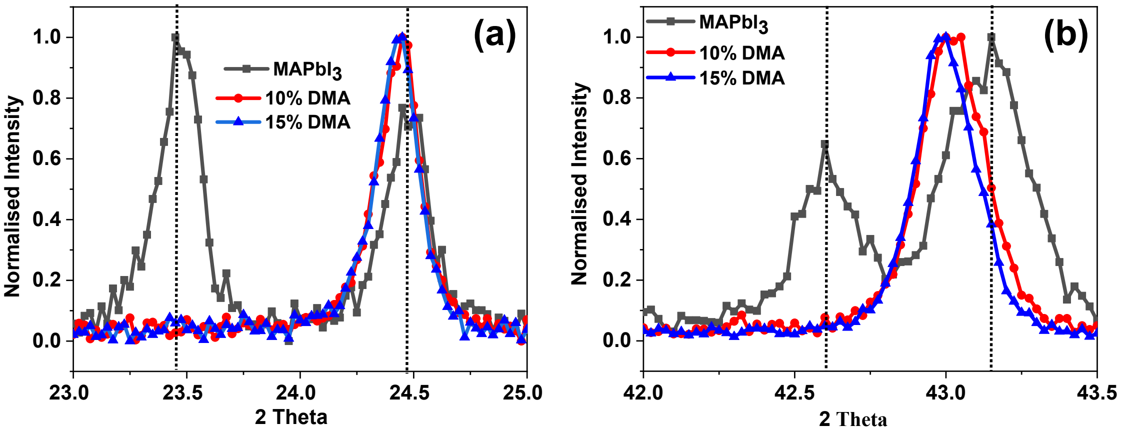


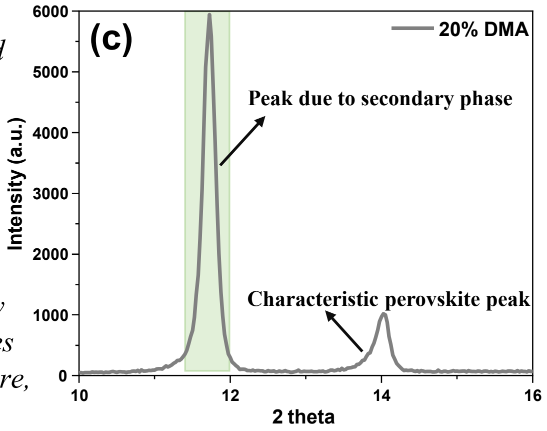


***Figure S3.*** *(**a** and **b**) Expanded diffraction data of DMA substituted composition (a-b), showing merged characteristic reflection planes (211) & (202), and (006) & (330) in DMA-substituted $MAPbI_3$, due to change in crystal symmetry from tetragonal ($MAPbI_3$) to cubic (10% DMA and 15% DMA). (c) Diffractogram showing diffraction obtained from a secondary phase of $DMAPbI_3$ [ref. [1] and references therein] in the case of 20% DMA films, which were, therefore, not considered further in this study.*

The thin film diffraction pattern of MAPI (Figure S2a) matches well with the tetragonal phase reported in the literature.[2,3] In the case of DMA, MA/DMA substitution is possible up to 15 at. % as seen from the diffractogram where larger d-spacings appear (Figure S2a). The 20 at.% DMA substitution results in secondary phase formation, therefore the study was limited to 15 at. % DMA substitution only (Figure S3). Here the substitution of DMA lowers the tetragonal to cubic phase transition temperature of $MAPbI_3$ as the DMA-substituted mixed perovskites are stable in the cubic phase even at RT as is evident from the absence of the characteristic (211) reflection in the of 23-25 2ϴ range (marked with red asterisk in Figure S3a). This is in line with literature reports, which show that even a small MA/DMA substitution of 5 at% changes the symmetry.[4–6] Also, the diffraction planes (006) and (033) at higher angles (□42°- 43° 2θ) merge to form single (003) plane in the 10-15 at% DMA film, which is evidence for a change in symmetry (Figure S3b).[5] Contrary to this behavior, in the films with MA/Gua and MA/AA, substitution was possible up to 20 at.% Gua and 15% AA with no change in crystal symmetry (Figure S3c).

**<u>Calculation of possible $DMAPbI_3$ (and $AAPbI_3$) on the surface of $MA_xDMA_{1-x}PbI_3$ ($MA_xAA_{1-x}PbI_3$):</u>**

Surface area = $4\pi r^2$  r=100 nm; surface area = $12.6 \times 10^4$ $nm^2$;

Unit cell dimensions for cubic MAPI = 0.639 nm (it will be slightly larger for $MA(DMA)PbI_3$ so to round off we use 0.64 nm).

Number of unit cells in the surface (one monolayer) = $12.6 \times 10^4 / (0.64)^2 = 31 \times 10^4$ ~$\mathbf{3 \times 10^5}$

Bulk volume = $4/3\ \pi r^3 = 4.2 \times 10^6$ $nm^3$

# of unit cells in 100 nm radius (200 nm diameter) sphere (~160 nm cube) = $4.2 \times 10^6 / 0.64^3$ = $\mathbf{16 \times 10^6}$

For nominally 15% DMA:

If we assume the bulk remains at 10% DMA (it actually is a little higher);

The extra 5% of DMA adds up to $1.6 \times 10^6$ DMA molecules

There will be $3 \times 10^5$ DMA molecules on the surface, assuming all the surface is $DMAPbI_3$ (probably slightly less due to the expected somewhat larger size of the $DMAPbI_3$ compared to MAPI),

i.e. total of ~$1.9 \times 10^6$ molecules DMA compared to $2.4 \times 10^6$ molecules DMA if a homogeneous 15% DMA solid solution were formed.

Therefore between 2 and 3 monolayers of $DMAPbI_3$ at the surface will be consistent with the (near) lack of lattice increase between the 10% and 15% DMA samples.

It is also possible that some $DMAPbI_3$ exists as small clusters (which will not show up in our XRD pattern).

Figure S3c shows a strong peak of $DMAPbI_3$ (see below) when 20% DMA was attempted, so it is reasonable to suggest that $DMAPbI_3$ may exist to a small degree in (nominally) 15% DMA, even though not seen in XRD. In fact, it is also possible that all the excess DMA is in the form of very small clusters. However, the formation of a very thin 2D layer on 3D perovskites is known to occur in some cases when a large A cation is present in the deposition solution.[7]

A similar argument can be made for the almost identical $2\theta$ positions of the 10% and 15% AA samples (and indeed, the small differences in position between the 15% and 20% Gua, where, though the broadened 20% Gua XRD peak at higher angle indicates a distribution of compositions).

## <u>Strain measurement in Gua and DMA substituted perovskite thin films using diffraction studies:</u>

Changes in lattice parameters  can be expected because of substitution with a bulkier cation that can lead to lattice strain.[8,9] $MAPbI_3$ serves as the reference composition in the strain measurement to which the other compositions are compared. Thus, any strain in the $MAPbI_3$ film is taken as a basis and we looked for changes in strain due to size in the mixed cation-substituted films. To calculate the strain we used Williamson-Hall (W-H) plots, derived from the FWHM of diffraction peaks presented in the respective diffraction data.[10] We could see an increase in peak broadening in all the substituted films, which can be due to crystallite size and/or strain (the instrument broadening was already accounted for). The W-H plot for all the substituted compositions are presented in Figure S4. The strain in Gua is more than in DMA-based films, when substituted more than 10%, and vice versa at 10% where Gua has almost no strain similar to $MAPbI_3$ ( Figure S4

and Table S2). The strain values calculated for various perovskite composition varies in the following order:

20%Gua □ 15%Gua >15% DMA> 10% DMA> 10%Gua ≥ 10%AA~$MAPbI_3$

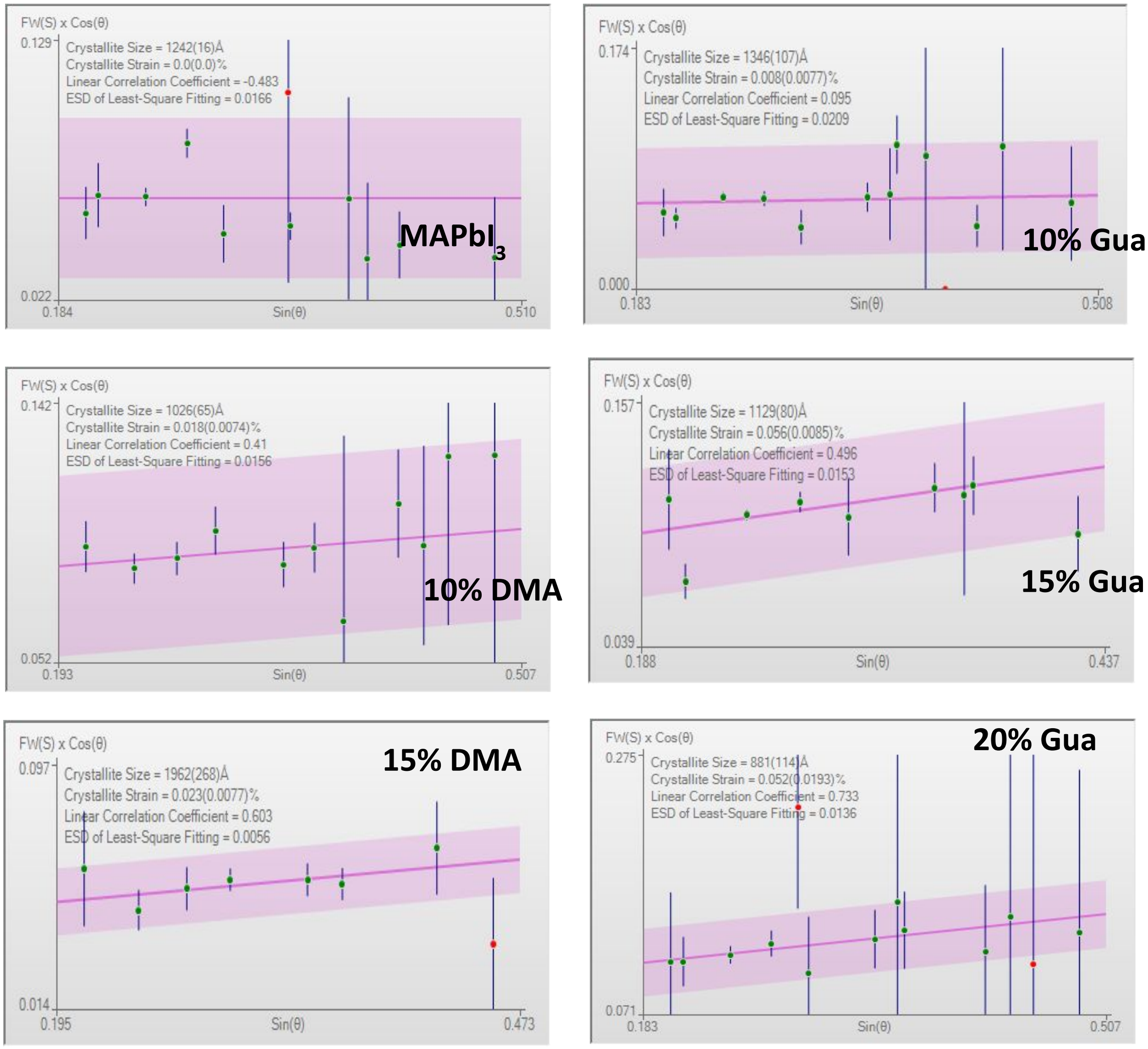


***Figure S4**. Strain and crystallite size calculations for the different $MAPbI_3$-based-thin films, with a fraction of $MA^+$ substituted with $DMA^+$ or $Gua^+$, using Williamson-Hall plots, obtained from the XRD measurements. The values of crystallite strain and size are given in Table S2, for each composition, with the errors in parentheses.*

The strain in all cases was calculated using the effect of both size and strain on peak broadening. The strain in $MAPbI_3$ is zero; therefore, its pink line is parallel to $\sin\theta$ on the x-axis of W-H plot, while in other cases we have a slope which gives the value of the finite strains inside the film. The strain in 10% Gua is almost zero, while 15% and 20% Gua films show the largest strains, more than twice that in the 10 and 15% DMA films.

***Table S2.*** *Compositions and their corresponding strain and crystallite sizes calculated using W-H plot obtained from XRD pattern. Errors are given in parentheses.*

| Composition | $MAPbI_3$ | 10% DMA | 15% DMA | 10% Gua | 15% Gua | 20% Gua |
|---|---|---|---|---|---|---|
| Strain | 0.0 | 0.018 (0.0074%) | 0.023 (0.0077%) | 0.008 (0.0077%) | 0.056 (0.0085%) | 0.052 (0.0193%) |
| Crystallite Size | 124 nm (1.6 nm) | 103 nm (6.5 nm) | 196 nm (27 nm) | 135 nm (11 nm) | 113 nm (8 nm) | 88 nm (11 nm) |

**H bonding from IR spectroscopy: Comparing the intensity of -NH band in different mixed Pb iodide perovskites-**

Based on the molecular structure of the cations, shown in Scheme 1 in the main article, it is expected that DMA can form 2 H-bonds (H - - I) per DMA compared to 3 such bonds for each MA (the difference being due to that DMA has an —$NH_2$ and MA and —$NH_3$ moiety). For Gua, 6 such H-bonds are possible per formula unit (due to the presence of three —$NH_2$ groups).[11] We used Polarization Modulation-InfraRed Reflection-Adsorption Spectroscopy (PMIRRAS) to study the H-bonding among considered compositions.

In the $APbI_3$ perovskites, the H in —NH bond of the organic A cation, is directly involved in the H-bonding with I of the $PbI_6$ octahedra. Therefore, we compare the strength (weak, w, medium, m, strong, s) of different —NH vibrational modes of the various A cations between the different perovskite compositions (Figure S5a). There is no shift in vibrational frequencies of the —NH band of -$NH_{3(MA)}$ upon partial substitution of MA by DMA or Gua (Table S3); possible reasons are the lack of direct interactions between neighboring A cations and the soft nature of the Pb-I matrix.

In the mixed DMA-MA compositions, the nature of the -NH bond in -$NH_{2\,(DMA)}$ and -$NH_{3\,(MA)}$ are similar due to the very similar chemical environments (N is $sp^3$ hybridized with localized positive charge, which makes N more electron-deficient and weakens the N-H bond). Therefore, the stretching and bending vibrational frequencies for -$NH_{2\,(DMA)}$ and -$NH_{3\,(MA)}$ in mixed DMA-MA and $MAPbI_3$ appear in the same range (Figure S5 a and Table S3).

However, the nature of -NH in $Gua^+$ is entirely different from that in DMA and MA. The $Gua^+$ cation has a single positive charge delocalized over 3 planar trigonal C=N bonds, which makes N less electron deficient and the corresponding N-H bonds will be stronger, and their vibrational modes will absorb at higher energy (Figure S5 a, b and c).[12]

The relative intensity ratio of stretching and bending modes of —$NH_{2\,(Gua)}$ and —$NH_{3\,(MA)}$ in Gua-MA mixed perovskite increases with increasing Gua fraction from 10 to 20% (Figure S5 b, c and Table S4). It is clear from various literature reports that H-bonding increases the peak area of the group that is involved in H-bonding.[13,14] Therefore, when the fraction of Gua increases from 10% to 20%, the intensity of the –NH band that accounts for sym. and asym. $NH_{2(Gua)}$ stretches also increases in the region ~3340-3460 $cm^{-1}$. Strong bending $NH_{2(Gua)}$ vibrations around 1656 $cm^{-1}$ behave similarly (Figure S5 b, c, and Table S3). However, the contribution of H-bonding is

decreased in the $NH_{3(MA)}$ stretching region which is due to substitution with different fractions of $NH_2$-containing Gua.

Similarly, reduction in H-bonding in case of DMA substituted perovskite has led to slight decrease in the peak area of the –NH band account for both sym. and asym. $NH_{2(DMA)}$ and $NH_{3(MA)}$ in the region ~3136 -3180 $cm^{-1}$ (Figure S5 a and Table S3).

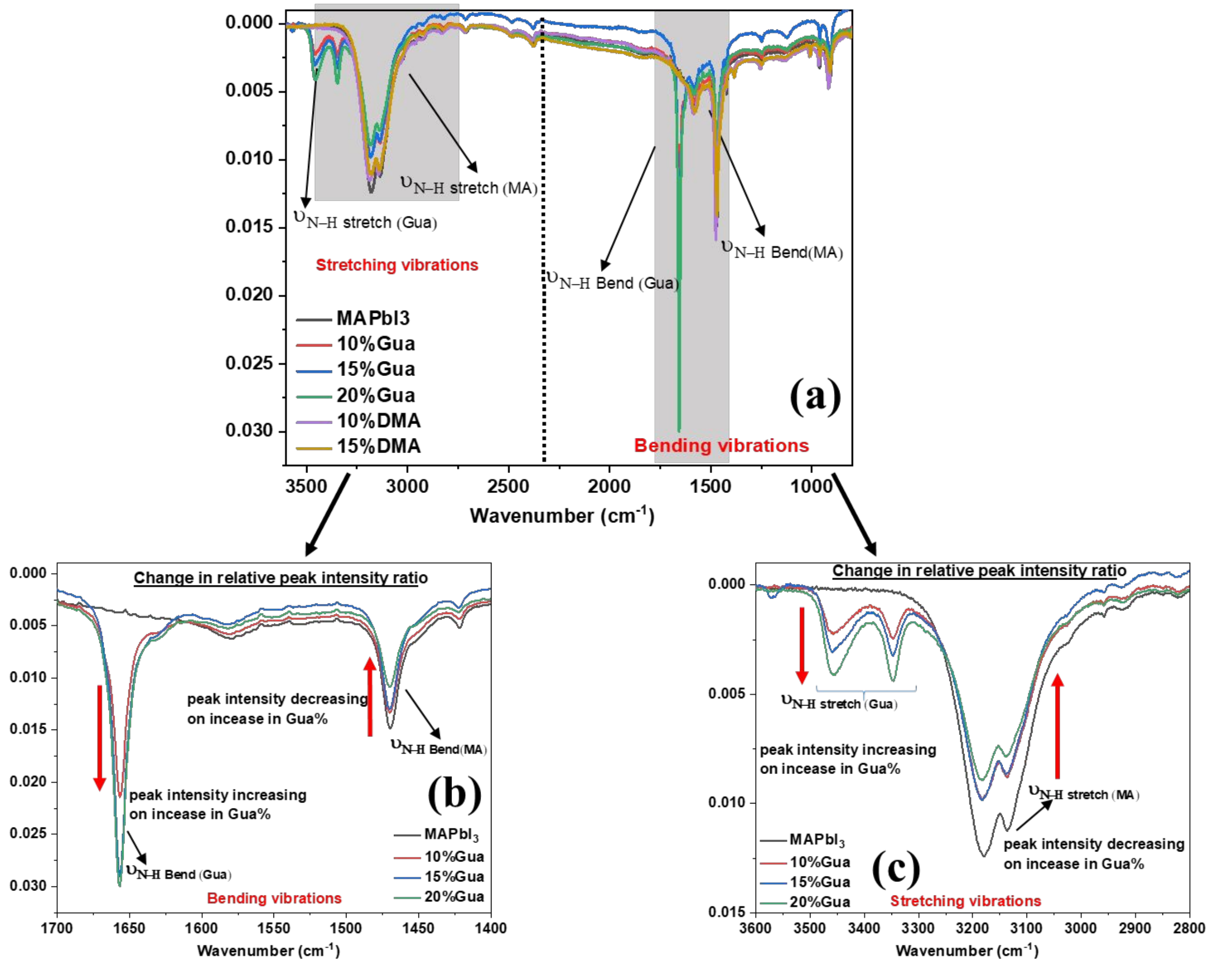


***Figure S5***. *(a) PMIRRAS spectra of the perovskite thin films substituted with different fraction of DMA⁺ and Gua⁺ cations. Attention has been given to strong -NH vibrational modes involved directly in H-bonding (shaded grey). (b and c) Enlarged view of Figure S5a showing variation in the relative peak intensity ratio of -NH bending and stretching vibrations, respectively, in Gua⁺-substituted perovskite, compared to $MAPbI_3$.*

***Table S3.*** *Comparison of peak positions (in $cm^{-1}$) of weak (w), medium (m) to strong (s) intensity - NH stretching and bending vibrations in $MAPbI_3$ thin films with different fractions of $MA^+$ substituted for by $DMA^+$ and $Gua^+$ cations.*

| *all frequencies in $cm^{-1}$* | **$MAPbI_3$** | **10% Gua** | **15% Gua** | **20% Gua** | **10% DMA** | **15% DMA** |
|---|---|---|---|---|---|---|
| **$CH_3$–$NH_3^+$ rock (m)** | 911 | 911 | 911 | 911 | 912 | 911 |
| **$CH_3$–$NH_3^+$ rock (w)** | 962 | 961 | 961 | 961 | 960 | 960 |
| **$CH_3$–$NH_3^+$ stretch** | | | | | 1007 | 1007 |
| **$CH_3$–$NH_3^+$ rock** | | | | | | |
| **$CH_3$–$NH_3^+$ bend (s)** | 1469 | 1470 | 1469 | 1470 | 1469 | 1469 |
| **$NH_3^+$ bend (very weak)** | 1579 | 1581 | 1581 | 1581 | 1579 | 1578 |
| **$NH_2^+$ bend (very s)** | - | 1656 | 1655 | 1656 | - | - |
| **sym. $NH_3^+$ stretch (s)**<br>**asym. $NH_3^+$ stretch (s)** | 3136<br>3179 | 3135<br>3181 | 3137<br>3183 | 3137<br>3183 | 3139<br>3180 | 3137<br>3179 |
| **sym. $NH_2^+$ stretch (m)**<br>**asym. $NH_2^+$ stretch (m)** | | 3346<br>3457 | 3347<br>3458 | 3346<br>3456 | | |

***Table S4.*** *Comparison of peak intensity ratios of —N-H band in —$NH_{2\,(Gua)}$ to the —N-H band in —$NH_3^+{}_{(MA)}$ considering stretching and bending vibration modes.*

| **Compositions** | **Stretching Vibrational Modes** | | **Bending Vibrational Modes** |
|---|---|---|---|
| | **sym. $NH_2^+$ stretch/ sym. $NH_3^+$ stretch** | **asym. $NH_2^+$ stretch/ asym. $NH_3^+$ stretch** | **$NH_2^+$ bend/ $NH_3^+$ bend** |
| $MAPbI_3$ | 0 | 0 | 0 |
| 10% Gua | 0.27 | 0.21 | 1.6 |
| 15% Gua | 0.48 | 0.41 | 2.10 |
| 20% Gua | 0.56 | 0.46 | 2.79 |

The values in Table S4 have been extracted from Figure S5b and c which show that as the fraction of Gua increases in the substituted perovskite, the peak intensity of —*N-H* band of —*$NH_{2\,(Gua)}$* increases irrespective of modes (either stretching or bending). Therefore, their ratios -$NH_{2\,(Gua)}$/-$NH_{3(MA)}^+$ increase. The ratio of $NH_{2\,(Gua)}$/-$NH_{3(MA)}^+$ stretching and bending intensities are lowest in $MAPbI_3$, which does not contain any $Gua^+$ cation, and highest in 20% Gua.

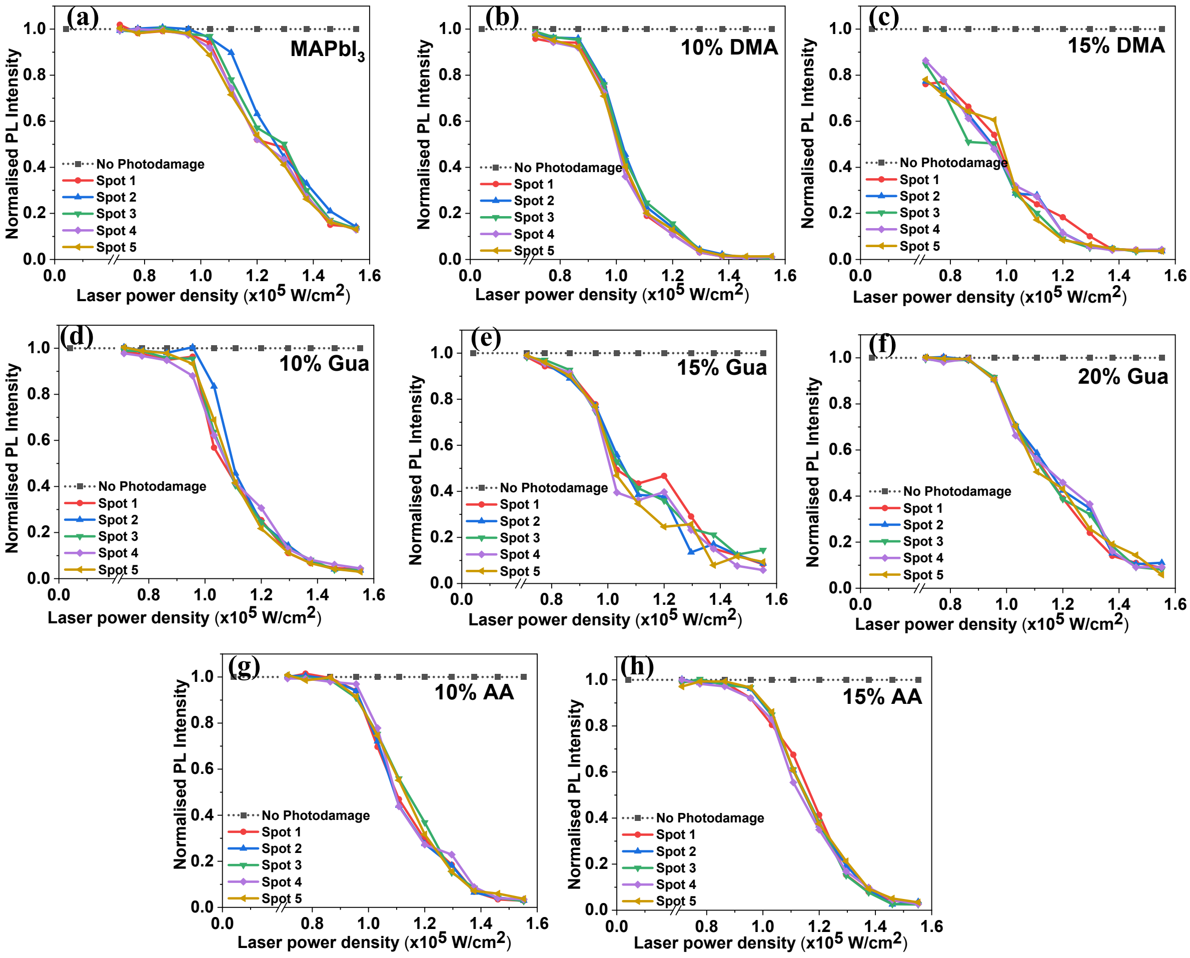


***Figure S6.*** *Various photo-damaging events have been created on different areas of films to check threshold variation. The figures present normalized photoluminescence emission intensity of encapsulated thin films substituted with DMA Gua and AA cations as a function of 488 nm laser power density of illumination. Data are shown for films of* ***(a)*** *$MAPbI_3$* ***(b)*** *10% DMA* ***(c)*** *15% DMA (d) 10% Gua* ***(e)*** *15% Gua* ***(f)*** *20% Gua* ***(g)*** *10% AA and* ***(h)*** *15% AA. For all samples, the same $4\times10^3$ W/cm$^2$ power density was used for scanning and the power density causing photodamage varied from 0.7 - $1.55\times10^5$ W/cm$^2$. The normalized PL intensities of the non-damaged area are given by the horizontal dotted black line.*

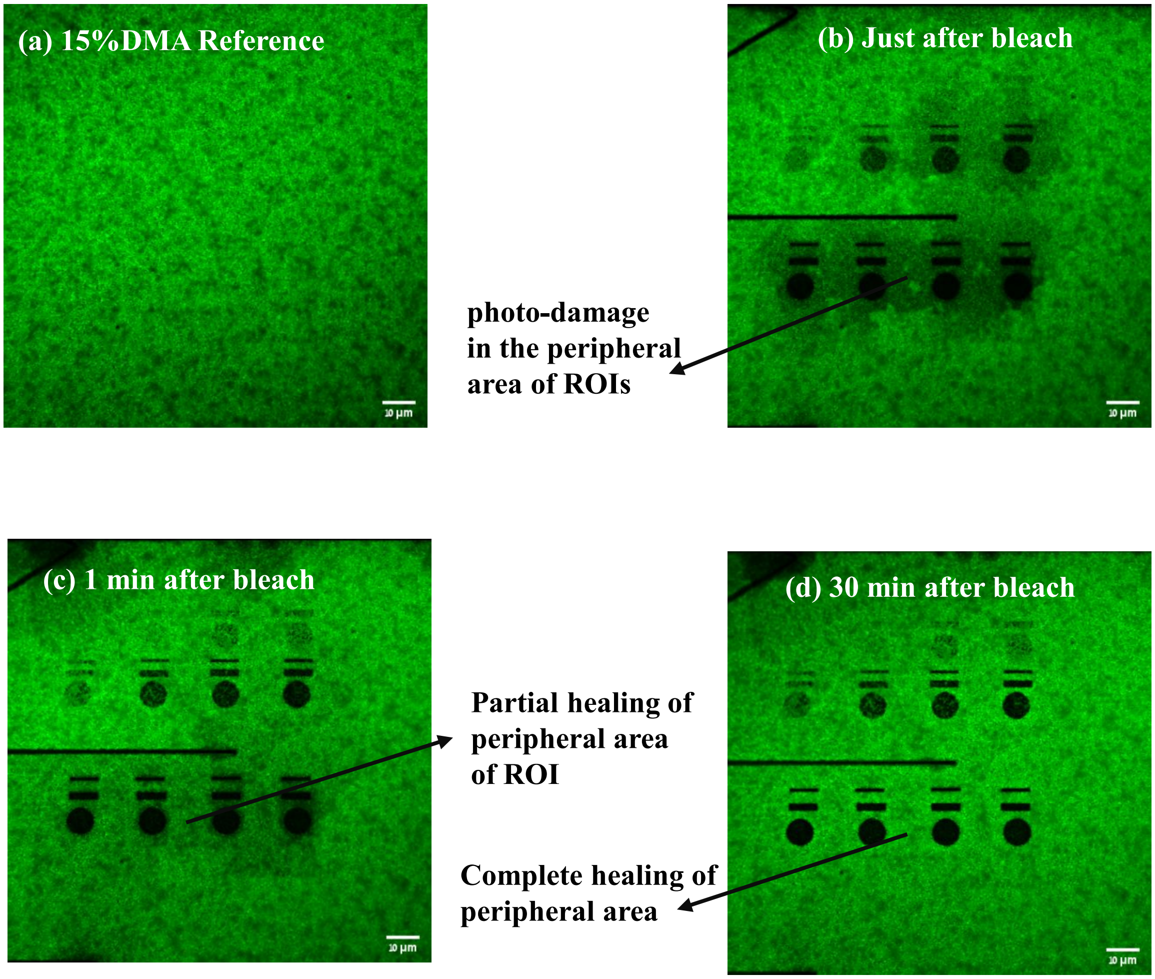


***Figure S7***. *Confocal microscope images of photodamage and recovery of areas beyond the ROIs onto which the photodamage was inflicted in 15% DMA films. (**a**) before photodamage, i.e., at t < 0; (**b**) Just after photodamage, i.e., at t = 0; (**c**) healed image after t = 1 min; (**d**) healed image after t = 30 min. It is important to note that such a phenomenon is absent in other substituted films., A more detailed description is provided in the main paper under the section 2.2 "Photodamage: function of the laser power density".*

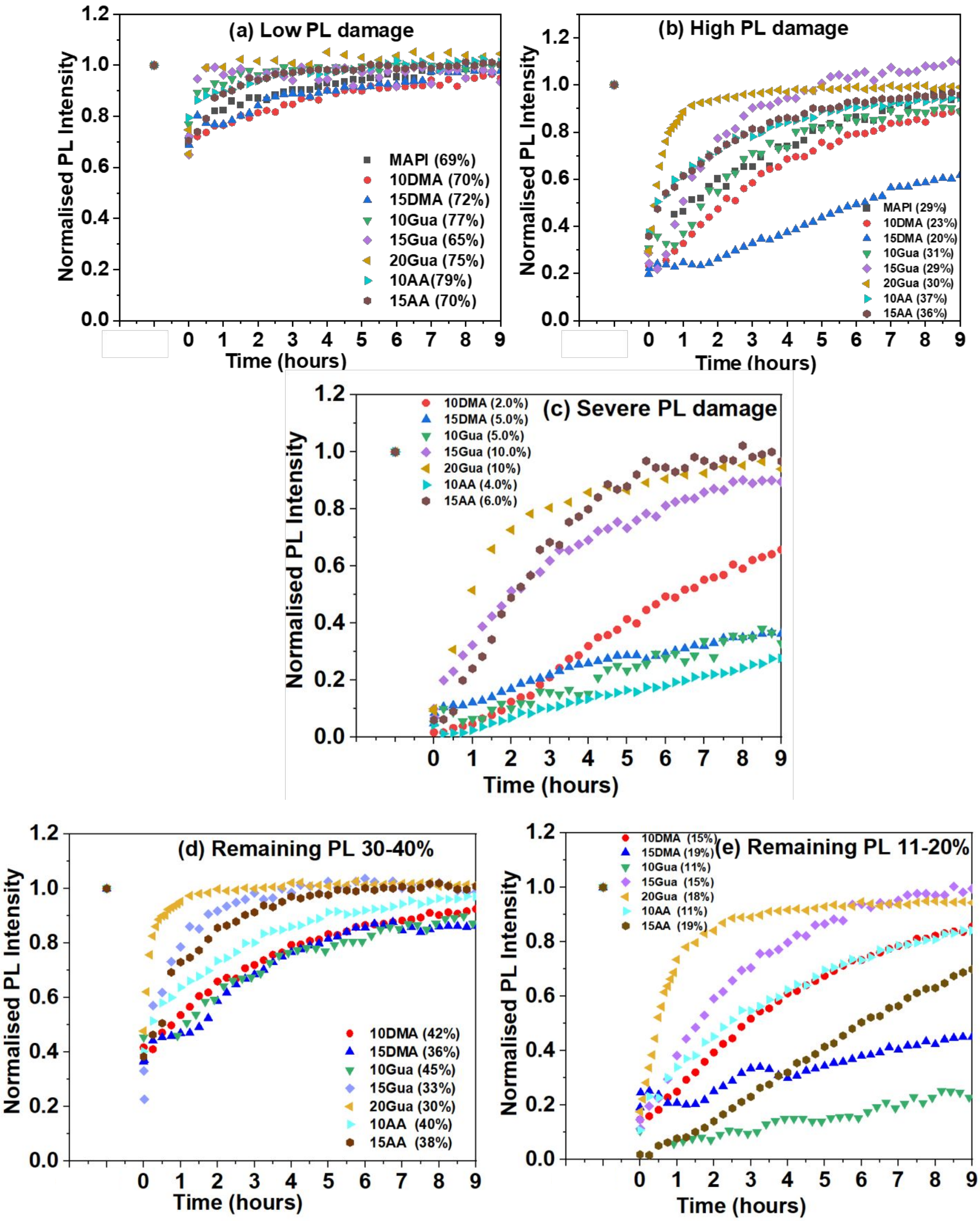


***Figure S8.*** *Additional self-healing kinetic plots for different degrees of damage.*

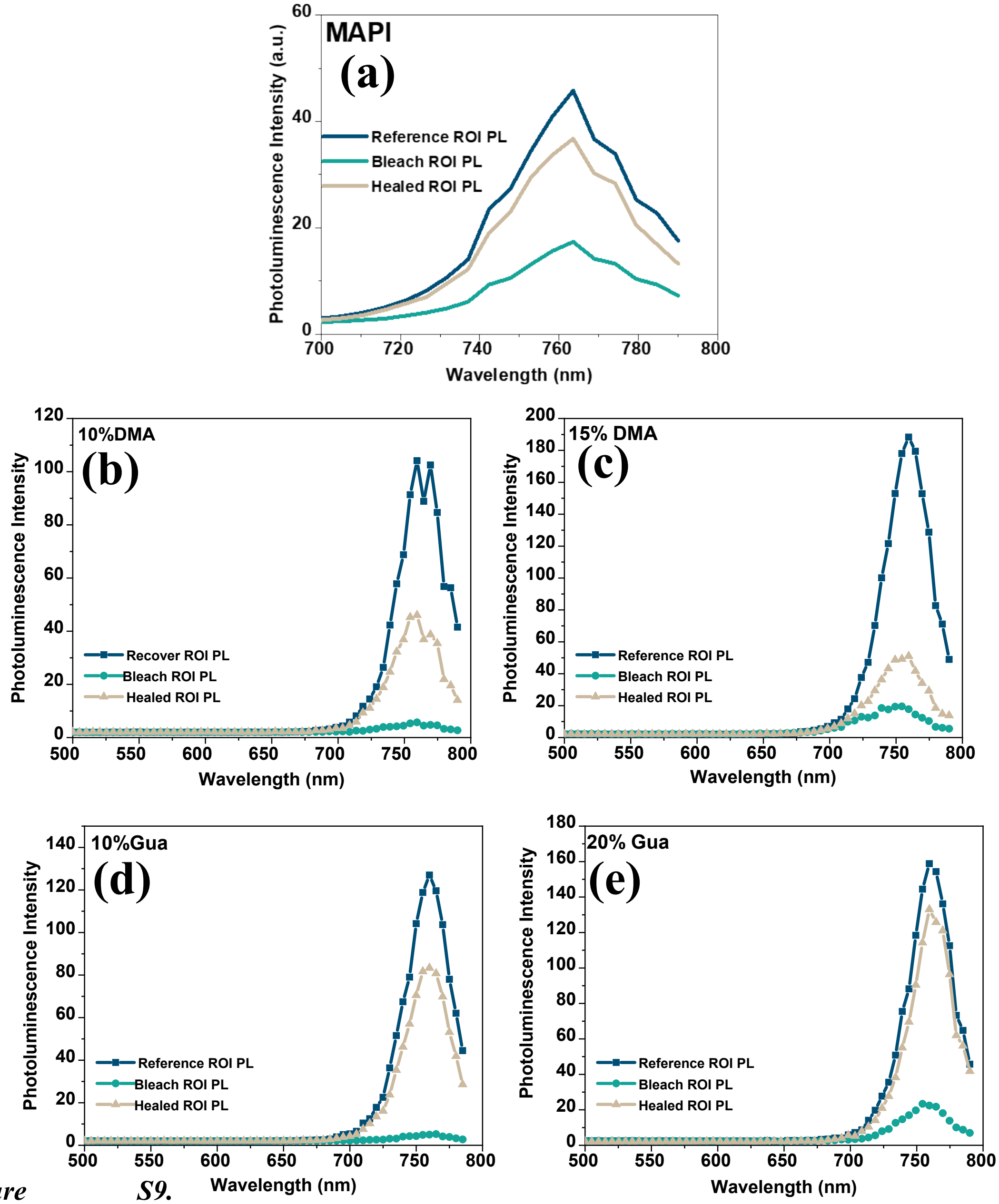


***Figure S9.*** *Photoluminescence spectra of encapsulated perovskite thin films with different fractions of $MA^+$ in $MAPbI_3$ substituted by $DMA^+$ and $Gua^+$ cations before and after photodamage, followed by self-healing. Spectra are recorded in the range 500-800 nm to show the absence of higher energy emission from a secondary phase that might form after healing.*

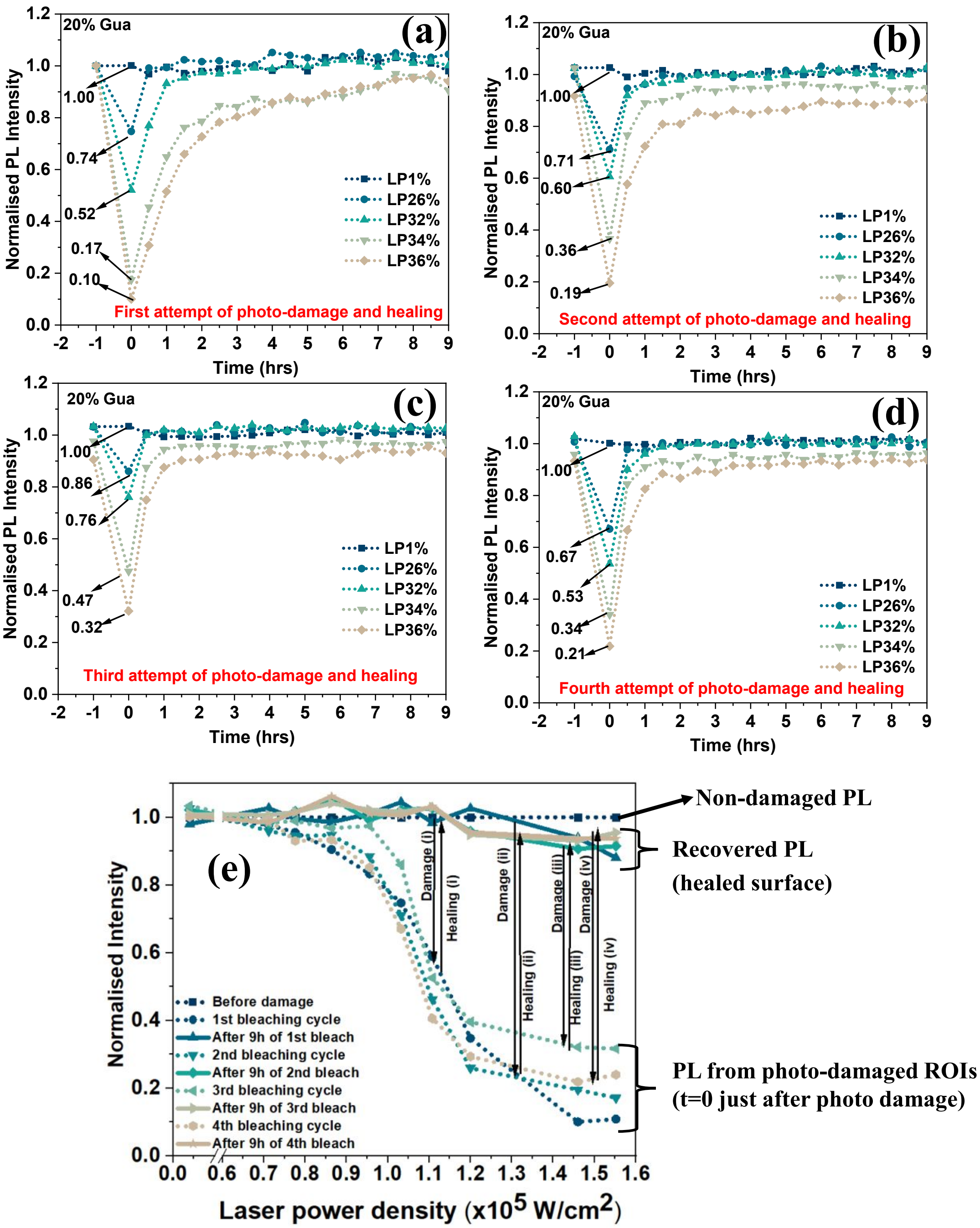


***Figure S10***. ***(a-d)*** *Four consecutive experiments of photo-damage and healing on the same ROIs (for each run; different ROI for each laser intensity) in a 20% Gua HaP film. Healing is fast which is characteristic of the 20% Gua films and occurs at a similar rate in all four photo-damage healing events.* ***(e)*** *Gua 20% healed >90% after every photo-damage event (grey horizontal dotted line at Y-axis with a value of ~1.0 refers to PL of non-damaged ROI; the dots below the full horizontal lines indicate the (reduced) PL of damaged ROIs and solid lines refer to normalized PL of healed ROIs); downward and upward arrows represent reversible damage and healing after each photodamage event.*

**Ease of Film formation:**

We carried out an experiment to investigate the ease of formation of the different samples. A visual inspection of MAPI, and DMA-and Gua-substituted MAPI films, immediately after the end of the spin-coating process, i.e, before solvent removal from the spin-coated film gave, reproducibly the results, shown in Figure S11. As can be seen from the results, DMA inhibits the formation of the perovskite phase and the partially substituted DMA films remain essentially colorless. At the same time, MAPI and partially Gua-substituted films give colored films closer to what is expected for the actual perovskite phases. We can postulate that it is the lower $\Delta G_{form}$ of the Gua-substituted material (than that of the DMS-substituted ones) that plays a role here.

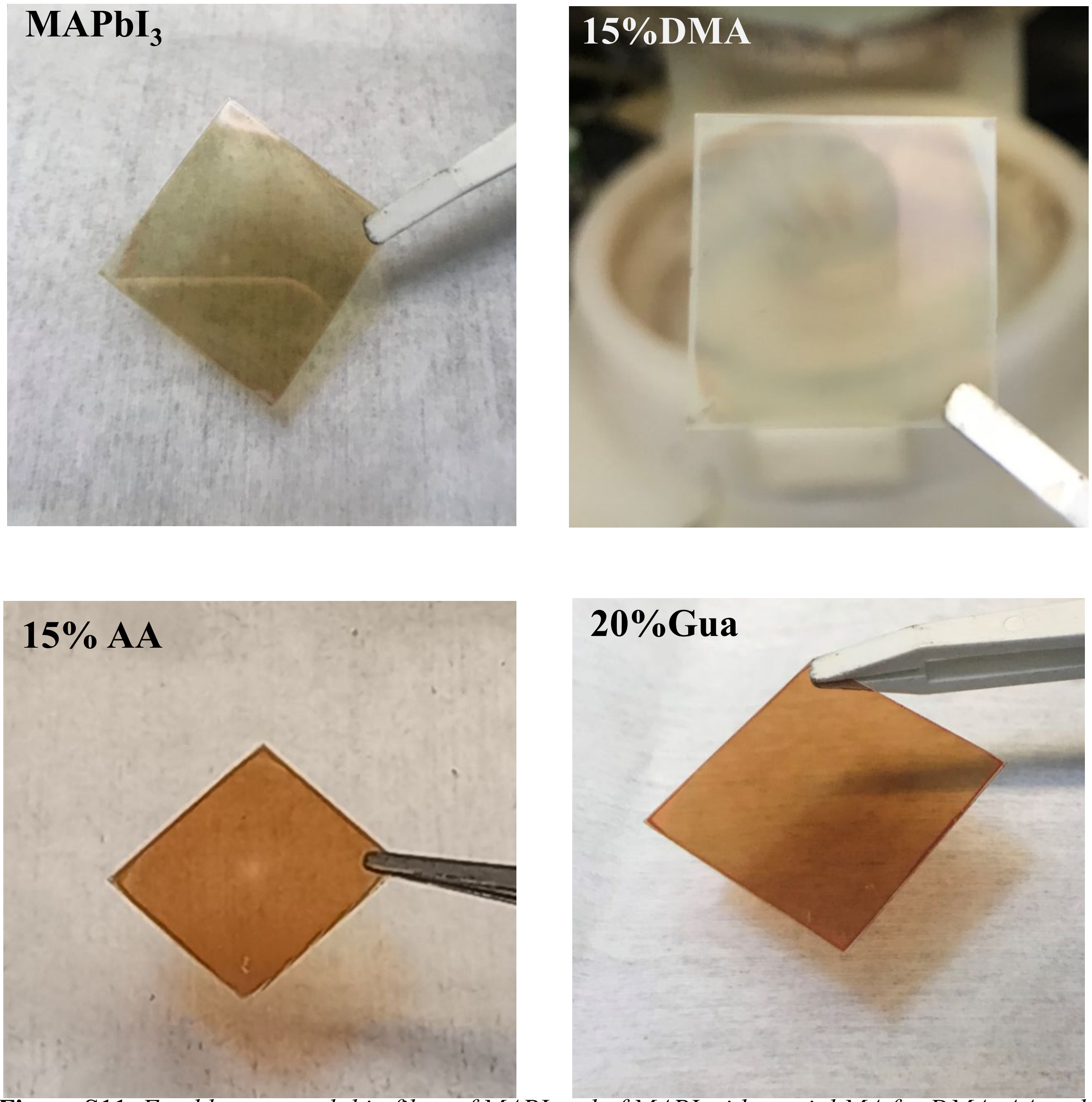


**Figure S11.** *Freshly prepared thin films of MAPI and of MAPI with partial MA for DMA, AA and Gua substitution, before thermal annealing.*

**References:**

(1) Pei, Y.; Liu, Y.; Li, F.; Bai, S.; Jian, X.; Liu, M. Unveiling Property of Hydrolysis-Derived $DMAPbI_3$ for Perovskite Devices: Composition Engineering, Defect Mitigation, and Stability Optimization. *iScience* **2019**, *15*, 165–172. https://doi.org/10.1016/j.isci.2019.04.024.

(2) Singh, P.; Soffer, Y.; Ceratti, D. R.; Elbaum, M.; Oron, D.; Hodes, G.; Cahen, D. A-Site Cation Dependence of Self-Healing in Polycrystalline $APbI_3$ Perovskite Films. *ACS Energy Lett.* **2023**, *8* (5), 2447–2455. https://doi.org/10.1021/acsenergylett.3c00017.

(3) Guo, X.; McCleese, C.; Kolodziej, C.; Samia, A. C. S.; Zhao, Y.; Burda, C. Identification and Characterization of the Intermediate Phase in Hybrid Organic–Inorganic $MAPbI_3$ Perovskite. *Dalton Trans.* **2016**, *45* (9), 3806–3813. https://doi.org/10.1039/C5DT04420K.

(4) Chen, H.; Wei, Q.; Saidaminov, M. I.; Wang, F.; Johnston, A.; Hou, Y.; Peng, Z.; Xu, K.; Zhou, W.; Liu, Z.; et al. Efficient and Stable Inverted Perovskite Solar Cells Incorporating Secondary Amines. *Adv. Mater.* **2019**, *31* (46), 1903559. https://doi.org/10.1002/adma.201903559.

(5) Franssen, W. M. J.; van Heumen, C. M. M.; Kentgens, A. P. M. Structural Investigations of $MA_{1-x}DMA_xPbI_3$ Mixed-Cation Perovskites. *Inorg. Chem.* **2020**, *59* (6), 3730–3739. https://doi.org/10.1021/acs.inorgchem.9b03380.

(6) Kong, W.; Ye, Z.; Qi, Z.; Zhang, B.; Wang, M.; Rahimi-Iman, A.; Wu, H. Characterization of an Abnormal Photoluminescence Behavior upon Crystal-Phase Transition of Perovskite $CH_3NH_3PbI_3$. *Phys. Chem. Chem. Phys.* **2015**, *17* (25), 16405–16411. https://doi.org/10.1039/C5CP02605A.

(7) Hu, J.; Wang, C.; Qiu, S.; Zhao, Y.; Gu, E.; Zeng, L.; Yang, Y.; Li, C.; Liu, X.; Forberich, K.; et al. Spontaneously Self-Assembly of a 2D/3D Heterostructure Enhances the Efficiency and Stability in Printed Perovskite Solar Cells. *Adv. Energy Mater.* **2020**, *10* (17), 2000173. https://doi.org/10.1002/aenm.202000173.

(8) Franssen, W. M. J.; Bruijnaers, B. J.; Portengen, V. H. L.; Kentgens, A. P. M. Dimethylammonium Incorporation in Lead Acetate Based $MAPbI_3$ Perovskite Solar Cells. *ChemPhysChem* **2018**, *19* (22), 3107–3115. https://doi.org/10.1002/cphc.201800732.

(9) Wu, J.; Liu, S.-C.; Li, Z.; Wang, S.; Xue, D.-J.; Lin, Y.; Hu, J.-S. Strain in Perovskite Solar Cells: Origins, Impacts and Regulation. *Natl. Sci. Rev.* **2021**, *8* (8), nwab047. https://doi.org/10.1093/nsr/nwab047.

(10) Liu, D.; Luo, D.; Iqbal, A. N.; Orr, K. W. P.; Doherty, T. A. S.; Lu, Z.-H.; Stranks, S. D.; Zhang, W. Strain Analysis and Engineering in Halide Perovskite Photovoltaics. *Nat. Mater.* **2021**, *20* (10), 1337–1346. https://doi.org/10.1038/s41563-021-01097-x.

(11) Stoddard, R. J.; Rajagopal, A.; Palmer, R. L.; Braly, I. L.; Jen, A. K.-Y.; Hillhouse, H. W. Enhancing Defect Tolerance and Phase Stability of High-Bandgap Perovskites via Guanidinium Alloying. *ACS Energy Lett.* **2018**, *3* (6), 1261–1268. https://doi.org/10.1021/acsenergylett.8b00576.

(12) Jodlowski, A. D.; Roldán-Carmona, C.; Grancini, G.; Salado, M.; Ralaiarisoa, M.; Ahmad, S.; Koch, N.; Camacho, L.; de Miguel, G.; Nazeeruddin, M. K. Large Guanidinium Cation Mixed with Methylammonium in Lead Iodide Perovskites for 19% Efficient Solar Cells. *Nat. Energy* **2017**, *2* (12), 972–979. https://doi.org/10.1038/s41560-017-0054-3.

(13) Cao, H.; Qi, F.; Liu, R.; Wang, F.; Zhang, C.; Zhang, X.; Chai, Y.; Zhai, L. The Influence of Hydrogen Bonding on N-Methyldiethanolamine-Extended Polyurethane Solid–Solid Phase Change Materials for Energy Storage. *RSC Adv.* **2017**, *7* (19), 11244–11252. https://doi.org/10.1039/C7RA00405B.

(14) Svane, K. L.; Forse, A. C.; Grey, C. P.; Kieslich, G.; Cheetham, A. K.; Walsh, A.; Butler, K. T. How Strong Is the Hydrogen Bond in Hybrid Perovskites? *J. Phys. Chem. Lett.* **2017**, *8* (24), 6154–6159. https://doi.org/10.1021/acs.jpclett.7b03106.
